# Comprehensive Study of Si-Based Compounds in Selected Plants (*Pisum sativum* L., *Medicago sativa* L., *Triticum aestivum* L.)

**DOI:** 10.3390/molecules28114311

**Published:** 2023-05-24

**Authors:** Aleksandra Orzoł, Edith Cruzado-Tafur, Adrian Gołębiowski, Agnieszka Rogowska, Paweł Pomastowski, Ryszard J. Górecki, Bogusław Buszewski, Małgorzata Szultka-Młyńska, Katarzyna Głowacka

**Affiliations:** 1 Department of Environmental Chemistry and Bioanalytics, Faculty of Chemistry, Nicolaus Copernicus University in Torun, Gagarina 7, 87-100 Torun, Poland; aleksandra@orzol.pl (A.O.); adrian.golebiowski@doktorant.umk.pl (A.G.); bbusz@chem.umk.pl (B.B.); 2 Department of Plant Physiology, Genetics and Biotechnology, Faculty of Biology and Biotechnology, University of Warmia and Mazury in Olsztyn, Oczapowskiego 1A, 10-720 Olsztyn, Poland; edith.cruzado@gmail.com (E.C.-T.); rigor@uwm.edu.pl (R.J.G.); 3 Centre for Modern Interdisciplinary Technologies, Nicolaus Copernicus University in Torun, Wilenska 4, 87-100 Torun, Poland; aga4356@wp.pl (A.R.); p.pomastowski@umk.pl (P.P.)

**Keywords:** analytical methods, antimicrobial properties, bioactive compounds, cytotoxic effects

## Abstract

This review describes the role of silicon (Si) in plants. Methods of silicon determination and speciation are also reported. The mechanisms of Si uptake by plants, silicon fractions in the soil, and the participation of flora and fauna in the Si cycle in terrestrial ecosystems have been overviewed. Plants of Fabaceae (especially *Pisum sativum* L. and *Medicago sativa* L.) and Poaceae (particularly *Triticum aestivum* L.) families with different Si accumulation capabilities were taken into consideration to describe the role of Si in the alleviation of the negative effects of biotic and abiotic stresses. The article focuses on sample preparation, which includes extraction methods and analytical techniques. The methods of isolation and the characterization of the Si-based biologically active compounds from plants have been overviewed. The antimicrobial properties and cytotoxic effects of known bioactive compounds obtained from pea, alfalfa, and wheat were also described.

## 1. Introduction

Silicon (Si) is the second most plentiful and stable element present in the Earth’s crust; it has a strong affinity for oxygen [1], and it is mainly present in the form of silicon dioxide (SiO_2_), quartz, and silicates. Silicon plays an important role in soil; it increases soil exchange capacity, enhances water and air patterns, and participates in the metabolization of phosphorus-containing minerals and the formation of aluminosilicates and heavy metal silicates (decreasing soil toxicity) [2]. Additionally, silicon is a beneficial mineral for plants regarding their growth, development, and environmental conditions [3], as it enhances the bioavailability of many beneficial macro- and micronutrients for plants [4]. Silicon participates in regeneration and acts against the negative effects of stress on plants (biotic and abiotic stresses), but the mechanism of this action is still not fully understood [1].

Silicon, mainly in the form of silica, can be taken up by the plant’s root system and transported along the plant’s various compartments (e.g., the structure of the cell walls), providing greater rigidity and flexibility, as well as utility values for agriculture [5]. Plants uptake Si in the form of monosilicic acid (H_4_SiO_4_) [3,6]; the transport is mediated through passive (transpirational stream) and active (from roots to shoots, mediated by specific transporter proteins) modes [3].

Plants produce a broad range of metabolites with diverse functions [7], such as alkaloids, steroids, tannins, glycosides, volatile oils, fixed oils, resins, phenols, and flavonoids, which are accumulated in different compartments of the cells [8]. The extraction of these bioactive compounds constitutes the first step in their characterization and utilization [7]. Si concentration in plants varies according to species, in a way that plants belonging to the Poaceae family and some species belonging to the family Fabaceae [9] show specific allocations of Si in all tissues (passive or active Si transport).

The main aim of extraction methods is to obtain the highest yield of the desired extract. Thus, the composition and concentration of the final extract will depend on the method selected. Some popular methods for extracting polar bioactive compounds from plant materials include maceration or percolation, microwave-assisted extraction (MAE) or solid-phase extraction (SPE), ultrasound-assisted extraction (UAE), and supercritical fluid extraction (SFE) [10]. Further, chromatographic analyses (such as thin layer chromatography, high-performance column liquid chromatography, gas chromatography, and so on) play a very important role in the identification of plant metabolic components, owing to several advantages, such as the specificity and possible use of such assays in qualitative and quantitative analyses [11]. Since bioactive compounds occur in plant material consisting of multicomponent mixtures, their separation, extraction, purification, and combination of chromatographic techniques are essential [12].

This paper describes the action of silicon in three plants belonging to the Fabaceae (especially *Pisum sativum* L. and *Medicago sativa* L.) and Poaceae (particularly *Triticum aestivum* L.) families regarding their physiology, strength, and utility for phytoremediation activities. Moreover, this review covers current analytical techniques, instruments, and methodologies used to understand the influence of silicon in plant development. Finally, it focuses on the isolation and characterization of the bioactive compounds in plant material, antimicrobial properties, and cytotoxic effects.

## 2. Silicon in Soil

**Figure 1:**
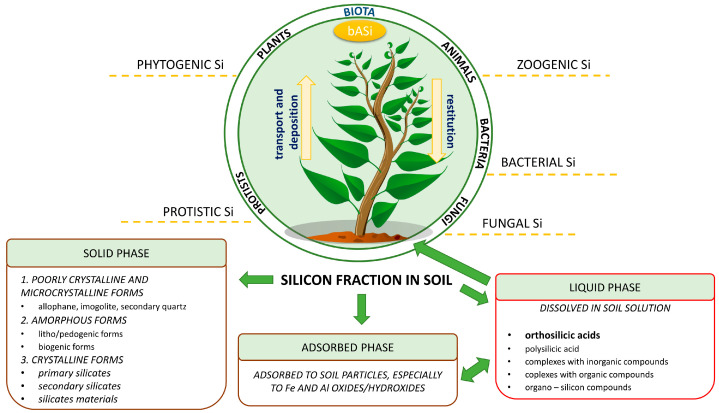
Schematic overview of the silicon fraction in soil and participation of biota in the Si cycle of terrestrial ecosystems, according to [16] and [18] with some modifications.

Si compounds in soil are usually present as silicon dioxide (SiO_2_), which comprises about 50–70% of the soil mass [13,14]. Other sources of Si include calcium and magnesium silicates, silicate slag, dolomite, rock phosphate, and diatomite [15]. Si compounds exist in the liquid phase, adsorbed phase, and solid phase of soil. In the solid phase, Si compounds can be divided into amorphous, micro-, poorly crystalline, and crystalline forms (Figure 1). The largest fraction of Si in the solid phase is crystalline forms, which occur as primary and secondary silica materials and silicates. Crystalline forms are poorly soluble and include feldspars, micas, clay minerals, and quartz [16]. The second most abundant fraction in the solid phase is amorphous forms originating from plant residues and remains of microorganisms (biogenic) or litho/pedonic materials [2].

The content of amorphous forms of Si in soils varies and usually ranges from 1 to 30 mg g^−1^ on a total soil basis. This amount depends on the plant cover, climate regions, and parental material [17]. The final forms of Si in the solid phase are poorly crystalline and microcrystalline, which include allophane, chalcedony, imogolite, and secondary quartz. The Si form available for plants in the soil (the liquid phase) depends on the solubility of Si in the solid phase. The liquid phase is comprised of dissolved Si, such as orthosilicic acid (H_4_SiO_4_) and polysilicic acid, and the polymerized and complexed silicic acid in the soil solution (complexes with inorganic and organic compounds or organo-silicon compounds) [18]. H_4_SiO_4_ is dissolved in the soil solution and can be adsorbed to soil mineral particles, especially iron and aluminum oxides/hydroxide, such as goethite and hematite. The total content of H_4_SiO_4_ in the adsorbed phase increases with pH and varies depending on the type of soil [19]. In general, the silicic acid content in the upper 20 cm soil layer contains only an average of 0.1 to 1.6 kg Si per ha as H_4_SiO_4_ [20]. The concentration of H_4_SiO_4_ can increase in the soil solution through the fertilization of farmlands where soils are inherently low in soluble silicon [19]. Silicic acid is also used by prokaryotes and eukaryotes in biosilification. The evolution of organisms revealed their capacity to use Si to synthesize siliceous structures. These organisms use H_4_SiO_4_ to synthesize hydrated amorphous silica, known as biogenic amorphous silica (bASi), in addition to other compounds [21].

Plant growth is dependent on the presence of various nutrients in the soil, which can be divided into essential, beneficial, and toxic elements [22]. Essential elements are necessary for plant growth, while toxic elements negatively affect plant development by disrupting various metabolic processes. Si, although present in plants, is not considered an essential element. Most plant species can complete their life cycle without it [23]. There is a lack of information on its physiological and biochemical role in plant biology [24]. However, various studies demonstrate that Si is beneficial for plant growth and development and in the mitigation of both abiotic and biotic stresses [14]. In addition, Si ions influence the increase in the availability of calcium, copper, manganese, molybdenum, phosphorus, sulfur, and zinc in the soil. Greger et al. [4] also concluded that chlorine and iron tended to increase, while potassium and magnesium were not much affected by Si.

## 3. Silicon Uptake by Plants

All plants grown in soil contain some amount of Si in their tissues. The Si concentration in plants ranges from 0.1% to 10% of dry weight. The concentration of Si in plant tissues depends on various factors, including the characteristics of Si uptake and transport. The Si uptake by plants varies between species and cultivars [25]. The Si content in plant tissues is high in monocotyledons, for example, in grasses (0.3–1.2% of dry weight) and very high in rice (up to 10%). Si taken up by plants is usually found in plant tissues as hydrogen-bound Si in organic complexes. The Si complex role is to impregnate the walls of the epidermis and xylem vessels, thus strengthening plant tissues and reducing water transpiration. It has also been demonstrated that Si associates with components of the cell wall (such as polysaccharides, lignins, and proteins) [26].

Silicon deficiency in soils can lead to negative impacts on intensive agricultural practices. The intensive cultivation of plants with high silicon accumulation (such as rice and sugar cane) in tropical areas significantly reduced soil plant-available Si (PAS). A silicon reservoir in soils can be maintained by phytoliths present in plants (amorphous SiO_2_), which are an important Si pool in the biogeochemical Si cycle. The recycling of silicon-rich crop residues, such as straw, is used to replenish the Si pool in the soil [27]. Among crop residues, rice straws (39%) and husks (24%) are the most abundant groups contributing to the total global return of Si phytoliths from crops. However, the use of plant residues only partially returns the Si taken up by plants to the soil [28]. Exogenous Si application significantly increases the Si content in the soil. Si is introduced into soils by the application of Si fertilizers (e.g., basalt powder) and biochar, which can induce the formation of phytolites and phytolith-occluded (PhytOC) organic carbon in plants [29]. Organic carbon can be occluded in 0.2% to 5.8% during the formation of phytolites. Dying plants introduce PhytOC into the soil, which becomes a reservoir of Si in the soil [30]. Appropriate application of Si fertilizers improves the bioavailability of nitrogen and phosphorus. Additionally, the soil application of Si has a positive effect on the diversity of microbial communities in the soil and their activity [31].

It is thought that the only form of Si taken up by plants is H_4_SiO_4_ [25], and the mechanisms of its uptake differ among plant species [14]. H_4_SiO_4_ is taken up as an uncharged monomeric molecule by roots from a soil solution below pH 9.0, and subsequently, it is transported through one of the possible mechanisms (passive or active) for the Si uptake to higher plants [32]. Silicic acid taken up by plants is not very mobile in plant tissues and has no electric charge [33]. The uptake mechanism of Si requires specific transmembrane proteins identified as Si transporters. The protein channels are passive transporters and do not require energy to transport Si across a cell membrane. In some plants (such as rice, barley, corn, wheat, banana, and cucumber), the transport of H_4_SiO_4_ through the plasma membrane is dependent on energy [34]. The first identification of Si transporters in monocot plants took place in rice. It was demonstrated that the mechanism of the Si uptake is faster than that of water. There is a specific system in rice roots facilitating the uptake of Si. This system facilitates silicic acid transport across the plasma membrane [35]. Silicic acid transport takes place using the Lsi1 (Low silicon rice 1) transporter, which belongs to a subfamily of aquaporin-like (NIP3). Lsi1 is located in the membranes of the main and lateral roots but not in the root hairs. This means that root hairs do not participate in the Si uptake from the soil solution [36]. H_4_SiO_4_ is taken up through the symplastic or apoplastic pathway and transported by the xylem to the shoots of the plant. It polymerizes into silica gel due to a loss of water (transpiration) and is deposited in different cellular compartments. However, Si in the xylem sap is present in the form of H_4_SiO_4_ [37]. Plants accumulate Si in their tissues to varying concentrations. On this basis, three categories of plants were distinguished: accumulators (rice, wheat, lentils, sugarcane, spinach, ferns, mosses, conifers) that accumulate more than 1.5% Si content in their tissues, intermediate accumulators (cucumber, soybean, pumpkins, rose, marigold, chrysanthemums) having Si in the range of 0.5–1.5%, and non-accumulators (tomato, grapes, sunflower, gerbera, petunia, begonia) which have a Si content below 0.5% [32,38].

## 4. Silicon Role in Plant Stress Alleviation

Si treatment mitigates many abiotic and biotic stresses, including heavy metal toxicity, salt, and drought (Table 1). Silicon can act by mechanical and biochemical or molecular mechanisms to aid plant growth. Plants deposit silicon in the form of spontaneous cell wall silicification, directed cell wall silicification, and directed paramural silicification in silica cells. Si modulates cell wall properties (thickness, rigidity, permeability, etc.) to adapt to the prevailing external conditions (silicification of the trichome and epiderma causes a higher ability to survive drought conditions; in exodermis and endodermis, it binds heavy metals), but Si also indirectly influences phytohormones, and production and activity of antioxidant enzymes [39]. However, the mechanism or mechanisms of these processes are still under investigation. In this review, the effect of Si-mediated stress alleviation in wheat, pea, and alfalfa plants is discussed.

## 5. Si-Mediated Alleviation of Stress Effects in Wheat

Wheat (*T. aestivum*) is a Si accumulator [34]. The Poaceae family, including wheat, can deposit Si as silica bodies (opal phytoliths) within particular cells of the leaf epidermis. Blackman [40] described the formation of silica cells in the sheath epidermis of wheat. During leaf growth, silica is accumulated and precipitated in the cells, appearing first as a translucent peripheral mass and later as glassy silica bodies containing small vesicles, which probably represent some remnants of the disintegrated cytoplasm [40]. The silica cells are located below the epidermis but also in the epidermal appendices, such as bristles or prickle hairs, and the shape of silica bodies is determined by the shape of the silica cell [41]. An analysis of the composition of silica bodies showed that it was near to stoichiometric SiO_2_ (41 *w*% silicon, 56 *w*% oxygen), and the SiO_4/2_ tetrahedrals were arranged preferentially in three-dimensional networks; a smaller proportion was in chains and layers. Moreover, the silica bodies with an overall amorphous structure contained crystalline precipitates, which could be indexed as α-quartz [41]. It was shown that the development of silicified trichomes in durum wheat depends primarily on the availability of Si in soil and is not affected by water stress. However, the accumulation of phytoliths over the veins provides better support to the leaf and ensures a better development of the whole plant [42]. Wheat plants can not only deposit Si in the aerial organs but also in the roots. Si impregnates the endodermis cell walls of the wheat seminal roots [43] and older parts of the root [44]. Si was detected in the large central metaxylem lumina in the basal zone of the root, in the smaller peripheral metaxylem and the immediately contiguous pericycle, and in the outer parenchyma cells bridging the small metaxylem vessels to the endodermal layer [44]. Casey et al. [45] identified two Si-containing species, mono- and disilicic acids (H_4_SiO_4_* and (HO)_3_Si(µ-O)Si(OH)_3_*) in a ratio of approximately 7:1, but no organosilicate complexes in the xylem exudate of wheat plants. Furthermore, after transferring plants to the solution containing 0.02 mM silicic acid and using the silica enriched to 98.7 atom percentage in ^29^Si, the Si concentration of the xylem exudate rises dramatically in a matter of minutes, indicating active mechanisms of Si transport across root cell membranes [45].

It was shown that different methods of Si application (soil treatment, foliar spray, and seed soaking) alleviated the Cd stress effects and significantly increased the wheat’s growth, chlorophyll fluorescence, photosynthetic gas exchange, water use efficiency, membrane stability index, relative water content, and the Si content [46]. However, among the three methods, Si applied as soil addition was the best and most effective in alleviating the Cd stress effects. Shi et al. [47] showed that when wheat seedlings were supplied with Si (through hydroponics), the treatment improved their biomass and photosynthesis but had little impact on the root morphology of plants grown under Cd stress. The application of Si nanoparticles made from sodium silicate (Na_2_SiO_3_) boosted the root morphology and physiology of wheat seedlings under Cd stress [48].

Moreover, amorphous silica (ASi) [49] and Si (K_2_SiO_3_) [50] decreased Cd concentrations in wheat shoots. The addition of Si (Na_2_SiO_3_) decreased Cd contents both in shoots and roots [51], and measurements of the Cd^2+^ flux showed that Si significantly inhibited the net Cd^2+^ influx in roots of wheat [47]. However, the study of Wu et al. [52] showed that Si did not accelerate Cd accumulation in cell walls or vacuoles. They concluded that Si decreased the Cd uptake by the increase in the root oxalate exudation as an avoidance mechanism. Greger et al. [50] showed that Si reduced Cd transport into the cytoplasm when Si was added both directly during the uptake measurements and to the growth medium. Silicate downregulated the expression of genes involved in the Cd uptake across the plasma membrane (i.e., LCT1) and the efflux of Cd into apoplast or vacuole from the cytosol (i.e., HMA2). Moreover, Si upregulated phytochelatin (PCS1) gene expression and enhanced the phytochelatin (PC) formation when Cd was present. The expression of iron and metal transporter genes (IRT1 and NRAMP1) was downregulated by Cd. However, the expression of IRT1 was upregulated by Si in root and shoot and NRAMP1 in roots only facilitating Fe transport in wheat [50].

Howladar et al. [46] showed that different Si application methods (soil treatment, foliar spray, and seed soaking) significantly reduced Cd^2+^ and increased the Si content. Si alleviated Cd toxicity in wheat seedlings by improving the antioxidant capacity and by decreasing the lipid peroxidation as a result of increased superoxide dismutase (SOD) and guaiacol peroxidase (POD) activity as well as decreased malondialdialdehyde (MDA) and hydrogen peroxide (H_2_O_2_) content in wheat leaves [47,51] and roots [53]. Howladar et al. [46] showed that the Si soil treatment, foliar spray, and seed soaking significantly alleviated the Cd stress in wheat and reduced the MDA content and electrolyte leakage, significantly increasing the content of proline and soluble sugars, antioxidative (POD, CAT, SOD) defense system activity, and the content of polyamines and their gene expression. Similarly, the incorporation of Si nanoparticles in a Cd-contaminated acidic nutrient solution increased the content of antioxidants and decreased the content of ROS in wheat roots compared with Cd single treatments in acidic pH [48]. The application of Si improved the water status of drought-stressed wheat plants [54]. The study of Pei et al. [55] also showed that the inclusion of Si in a culture solution maintained the leaf water potential of polyethylene glycol (PEG) stressed plants at the same level as that of the control plants. The addition of Si partially improved the growth of the shoot (but not the root) and increased the leaf chlorophyll concentrations of drought-stressed plants [55].

Moreover, compared with the non-Si treated wheat plants, the application of Si increased the activity of SOD, CAT, and glutathione reductase (GR), the fatty acid unsaturation of lipids, and the contents of photosynthetic pigments and soluble proteins, as well as total thiols, whereas the content of H_2_O_2_, the activity of acid phospholipase (AP), and the oxidative stress of proteins were decreased by applying Si [54]. The activity of glycolate oxidase (GO), POD, and ascorbate peroxidase (APX) showed no significant difference between under-drought and drought-Si treatment [54]. At the same time, Pei et al. [55] showed that PEG stress-induced membrane lipid peroxidation, as well as the decrease in the glutathione concentration, could be partly alleviated by adding Si. These authors [55] suggested that under short-term, PEG-induced water stress conditions (1 week), antioxidant defense rather than osmotic adjustment contributed to the improved wheat growth using Si.

The ability of Si to mitigate salinity stress (NaCl application) was also investigated in wheat [56]. Alzahrani et al. [56] tested Si roles in improving salt, drought or Cd stress tolerance in wheat. They showed that under stress conditions, the Si supplementation conferred higher growth, gas exchange, tissue water and membranes stabilities, K^+^ content, and limited MDA and Na^+^ contents and electrolyte leakage relative to those obtained without Si. Compared to those results obtained without Si, enzyme (e.g., SOD, CAT, and PO) activity was improved by Si applications, which was linked to elevated contents of antioxidants and osmoprotectants (e.g., free proline, soluble sugars, ascorbic acid and glutathione). The level of 4 mM Si was most effective in mitigating the salt and drought stresses, while 6 mM Si level was most influential to alleviate the Cd stress [56].

Additionally, Silva et al. [57] showed that supplying Si to wheat plants could increase the resistance to leaf streak caused by *Xanthomonas translucens* pv. *undulosa*, possibly through an increase in tissue lignification and the participation of chitinases and peroxidases. Moreover, Si-treated wheat plants had a clear adverse effect on the greenbug, *Schizaphis graminum* (Rond.) development due to possible Si-mediated changes in the quality of phloem sap [58].

## 6. Si-Mediated Alleviation of Stress Effects in Pea Plants

Grasses accumulate greater amounts of silica than other species of flowering plants. Parry and Winslow [59] investigated the accumulation of Si in seedlings of peas (*P. sativum* L.). Species that do not accumulate Si have no leaf deposits of phytoliths. However, Si was observed in vascular bundles and epidermal cell walls, often in close proximity to stomatal complexes but mostly in the tendril tips [59].

The beneficial effect of Si on the yield and nutrient uptake of peas was shown under sandy soil conditions [60]. There are also several studies that investigate whether and how Si influences the alleviation of heavy metal toxicity in pea plants [61,62,63]. Supplementation with Si reduced Cd accumulation and enhanced the uptake of macronutrients and micronutrients in shoots and roots, which depressed Cd toxicity in pea plants [63]. Rahman et al. [62] showed that the addition of Si in Cd-stressed plants noticeably increased growth and development along with the improved total protein and membrane stability of Cd-stressed plants. Moreover, Si supplementation modulated the total chlorophyll, carotenoid, photosynthetic efficiency, photochemical quenching, leaf relative water content, and gas exchange parameters in pea plants [63]. Rahman et al. [62] showed that GSH1 (phytochelatin precursor) and MTA (metallothionein) transcripts were predominantly expressed in roots and strongly induced due to Si supplementation in Cd-stressed plants compared with Cd-free conditions. This suggests that these chelating agents may bind to Cd, leading to vacuolar Cd sequestration in roots.

**Figure 2:**
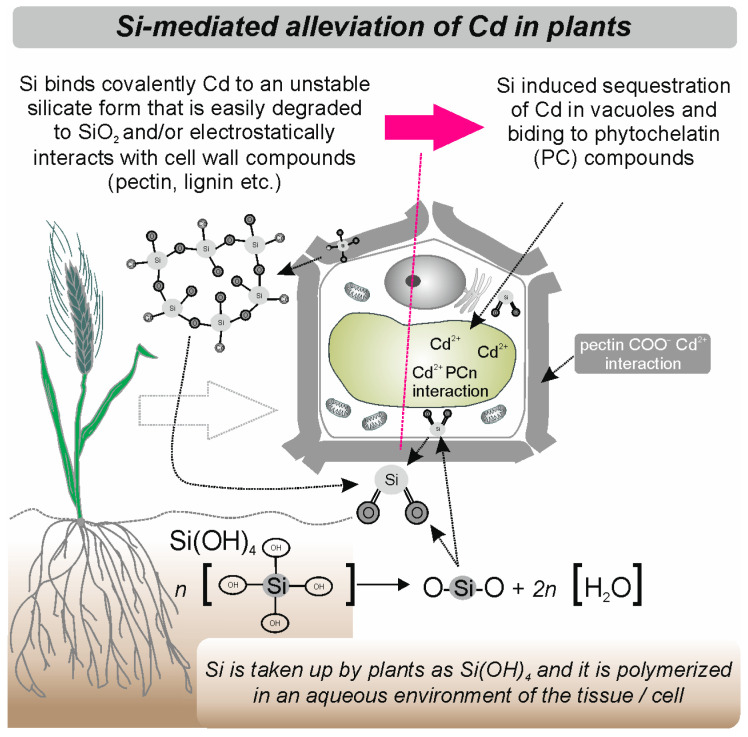
Transformations of Si in plant cells and mechanisms of alleviating stress caused by Cd. Adapted from Głowacka et al. [67] according to [5,68].

**Figure 3:**
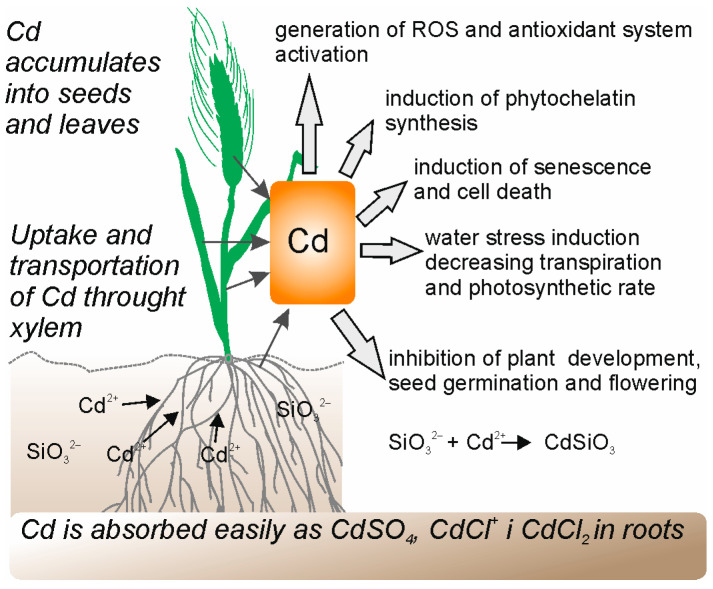
Diagram showing Cd uptake, transport, and accumulation along with selected Cd toxic effects on plants. Adapted from Głowacka et al. [67] according to [69,70].

Furthermore, pea Fe transporter (RIT1) showed downregulation in shoots when plants were treated with Si along with Cd compared with Cd-treatment conditions (Figure 2 and Figure 3). In conclusion, Rahman et al. [62] suggested that the alleviation of Cd toxicity in pea plants might be associated with Cd sequestration in roots and reduced Cd translocation in shoots through the regulation of Fe transport. Additionally, the effect of Si on Cd-stressed plants showed the involvement of the antioxidant defense system. The activity of CAT, POD, SOD, and GR increased along with elevated S-metabolites (cysteine, methionine, glutathione), implicating the alleviation of Cd toxicity in peas [62]. Furthermore, Si supplementation significantly decreased the antioxidant levels: the accumulation of H_2_O_2_, MDA content, electrolyte leakage, and methylglyoxal content [63]. It was also shown that the addition of silicon nanoparticles (SiNp) along with chromium (Cr (IV)) protects pea seedlings against Cr(VI) phytotoxicity by reducing Cr accumulation and oxidative stress and up-regulating the antioxidant defense system and nutrient elements [61]. Interestingly, Feng et al. [64] show that extracellular silica nanocoat formed by layer-by-layer (LBL) self-assembly confers aluminum resistance in root border cells of peas. Moreover, seed priming with Si also showed an effect on drought-stressed pea plants [65]. Si treatment improved the morphological, physio-biochemical, and yield characteristics of pea plants, such as chlorophyll a, chlorophyll b, and relative water content, regulated the up-regulation of antioxidant enzymes, increased seed yield, and decreased lipid peroxidation and reactive oxygen species, mainly superoxide and hydrogen peroxide, in drought-stressed pea plants [65]. Additionally, it is suggested that the Si application and accumulation within the plant may activate the host defenses (the increased activity of the enzymes chitinase and β-1,3-glucanase in leaf extracts) and the subsequent resistance to the fungal pathogen (*Mycosphaerella pinodes*) in pea plants [66].

## 7. Si-Mediated Alleviation of Stress Effects in Alfalfa Plants

The effect of Si application on growth, physiology [71] and the alleviation of stresses was investigated in alfalfa plants [72,73,74,75], Liu and Guo [71] showed that Si application (K_2_SiO_3_) alone increased the alfalfa (*Medicago sativa* L.) forage biomass and the number of branches. Moreover, the Si application reduced both the transpiration rate and stomatal conductance but had no effect on the photosynthetic rate. On the other hand, this promotive effect of the Si application on biomass and water use efficiency was regulated by the soil moisture conditions [71].

Kabir et al. [72] showed that the addition of Si in Cd-stressed plants caused a significant improvement in morpho-physiological features along with total protein and membrane stability. Furthermore, Si supplementation in Cd-stressed plants showed a significant decrease in the Cd and Fe concentrations in both roots and shoots compared with Cd-stressed plants, revealing that Si-mediated tolerance to Cd stress was associated with Cd inhibition in alfalfa. However, the mechanism of this process was to be established since no significant changes in the expression of two metal chelators (phytochelatin synthase and metallothionein) and PC accumulation after the Si application under Cd stress were observed. Interestingly, Kabir et al. [72] showed the Si-mediated alleviation of Cd toxicity in alfalfa limited the Fe uptake through the down-regulation of Fe acquisition mechanisms. Moreover, the increase of the activity of antioxidant enzymes (CAT, SOD, ascorbate peroxidase) and elevated methionine and proline content after the Si application may reduce H_2_O_2_ and provide defense against Cd stress in alfalfa [72].

The Si-mediated effect on salt stress tolerance involved the significantly increased plant biomass, nodules number, and N content in alfalfa plants [75]. Moreover, chlorophyll content, relative water content, predawn leaf water potential, water use efficiency, and relative water content increased after the Si application under salt stress [74,75]. Additionally, the concentration of Na^+^ was reduced by Si treatment with an increase in the K^+^ content [75] and increasing K^+^/Na^+^ radio to protect the leaves from Na^+^ toxicity [74]. The Si application induced the activity of antioxidant enzymes (SOD, CAT, POD) and decreased the MDA content [74]. Si treatment lowered the amounts of MDA and H_2_O_2_ and also reduced electrolyte leakage in salt-stressed alfalfa plants, but at the same time, increased total polyphenol, flavonoid, and carotenoid contents [75]. Moreover, compatible osmolytes (proline, glycine betaine, and soluble sugars) were found to have increased particularly after the Si treatment in comparison to Si-untreated plants. It is important to underline that alfalfa varieties reacted differently to the Si treatment [75].

Alkaline stress is one of the abiotic stresses limiting plant growth because of higher pH. Liu et al. [73] showed that the Si priming of alfalfa seedlings significantly alleviated the damage symptoms and increased biomass, chlorophyll content, photosynthesis, and water use efficiency. Moreover, the Si alleviated oxidative damage caused by alkaline stress (25 mM Na_2_CO_3_, pH 11.2) by decreasing membrane injury and MDA content and increasing POX and CAT activity in alfalfa leaves. In contrast with the Si effect on saline (120 mM NaCl) stressed plants, the Si priming significantly decreased the accumulation of protein and the proline content in alfalfa under alkaline stress [73]. Furthermore, they showed that Si-treated plants under alkaline stress accumulated more Na, Mg, Fe, Mn, and Zn in the roots but accumulated more K and less Na in the leaves [73].

## 8. Extraction Methods to Assess the Presence of Silicon in Plants

The presence of Si has beneficial effects on the crops such as sugar cane, rice, tomato, cucumber, and strawberry; hence, several procedures for the determination of the Si available in plants (e.g., amorphous silicon or total silicon in plant material) were developed [16]. The extraction methods were designed in view of providing a stable and reproducible approach, improving the extraction efficiency and selectivity. It consisted of the application of an anion to replace adsorbed Si, an effect that has been proven between the Si in the extract and the Si in the crop yield [16].

## 9. Sample Preparation and Si Concentration in Plant Material

Specific analytical techniques entail specific sample preparation protocols, considering their requirements [76], and silicon could be detected and quantified directly—in solid plant material (XRF, for example) or indirectly—after plant digestion into the aqueous phase (ICP technique, in particular) (Table 2). Plant material should be dried and fragmented to obtain homogenous material [77]. Techniques that are based on the liquid sample introduction system (most of the ICP techniques) require the dissolution of a sample to the liquid phase while reducing the occurrence and effects of the matrix on the results of the analysis [78]. Both solubilization and reduction of the matrix are achieved by pyrolysis [79], ashing [80], and digestion techniques; they involve using an oxidant reagent to obtain complete dissolution [81]. Barros et al. [82] performed two steps of a microwave-assisted digestion procedure of plant material with diluted reagents, firstly in HNO_3_ and secondly in NaOH, in order to reduce the matrix effect in the ICP-OES quantification. However, these techniques often require the use of toxic reagents that cause corrosion of the analytical equipment; therefore, procedures are being developed based on less toxic solvents [83]. Silicon can be dissolved by using a high concentration of alkali (Na or K-hydroxides) [84], acids (preferably HF) [85], and sodium carbonate solution [86].

These procedures are dedicated to the elemental analysis of material; they are associated with the dissolution of silicon species to ionic form Si^4+^ (aqua, ligands). The crucial factor is the presence of fluoride anions in the solution due to their solubilizing (silicon creates a compound with fluoride more stable than oxygen) and stabilizing properties of silicon in the solution, especially at the trace concentration level [93]. Saito et al. [94] reported that extraction with a mixture of HF (1.5 M HF and 0.6 M HCl) could dissolve 150 mg SiO_2_ within 1 h of extraction from 500 mg of a rice plant sample. The volume of HBF_4_ to dissolve 100 mg of sample was 0.2 mL of 48% (*v*/*v*) [95]. Taber et al. [87] compare using the mixture of acid (HCl/HF) with autoclave-induced digestion (AID). The AID method may be useful owing to the rapid determination of Si in plants without using HF, but it is not suitable for crops with a low Si concentration. The comparison of HCl/HF extraction with AID for selected leaf reference materials indicated good agreement for corn stalks and bluegrass clippings but not for apple or peach leaves. He et al. [96] used a mixture of nitric acid and hydrogen peroxide to sample preparation for silicon concentration determination from the cell wall of a rice plant.

Nowadays, modern laboratories use methods of sample preparation based on microwave-assisted mineralization [97]. This technique is based on the synergy of three features: high pressure and temperature along with the action of concentrated acids, which ensure very good recovery, reproducible results, and rapid analyses without a greater risk of sample contamination. The mixture of nitric acid (a very good solubilizing agent for most elements, which has the oxidation ability towards organic matter), hydrofluoric acid (the reason for it being applied is described above), and the same volume of water in the digestion procedure is followed by the second digestion step: the neutralization of excess HF by using boric acid (due to highly corrosive and toxic properties of HF) [81,98]. Sometimes, adding an additional oxidant reagent is helpful [99,100]. Plant material is very rich in organic carbon, and it is necessary to enhance the oxidative power of the solvent to reach complete digestion. Cross-contamination should be avoided, especially with glass materials, etc. (containing silicon), when working with HF for a quantitative analysis of silicon. Silicon can be bound to aluminum species, which makes its extraction more complicated [101].

The ashing and melting of plant material with alkaline flux constitute another possible approach to preparing samples for silicon determination. Silicon dioxide is not a volatile compound; thus, there is no risk of silicon loss. Bowen et al. [102] carried out the ashing method on plant material at a maximal temperature of 500 °C for 10 h, and next, the ash was fused with anhydrous Na_2_CO_3_. Pan et al. [103] prepared samples of rice plants by ashing the material at 550 °C for 3 h and then dissolving the ash in diluted hydrogen fluoride.

Raid et al. [104] presented a method based on gravimetric determination of silicon in plants as SiO_2_. After the pretreatment, a plant sample was digested in a mixture of 5 HNO_3_:H_2_SO_4_:2 HClO_3_ and undissolved parts were filtered away; then, the sample was ignited and ashed in a porcelain crucible. The residue is SiO_2_, determined by the mass measurement of the silicon content [104].

## 10. Extraction Methods to Assess the Presence of Plant Biologically Active Compounds

The initial stage is to carefully prepare samples in order to preserve the biomolecules synthetized by plants; fresh and dried samples (leaves, barks, roots, stems, fruits, and flowers) could be used, but considering that a smaller particle size increases the surface contact between samples and extraction solvents, it is important to powder the plant material; it can be previously air-dried, microwave-dried, oven-dried, or freeze-dried (lyophilization) [105]. Pre-treatments are expensive and have limited influence on the process of converting biomass into target compounds, which is why it is still necessary to improve their efficiency and reduce costs [106].

The purpose of extraction techniques is to separate soluble plant metabolites, so that the initial crude extracts contain a complex mixture of plant metabolites, such as alkaloids, glycosides, terpenoids, phenolics, and flavonoids (Table 3). Extraction is an important step in the analysis of plants, being necessary to extract components for further separation and characterization, using an adequate solvent, such as methanol, ethanol or ethyl-acetate, dichloromethane, or a mixture of dichloromethane/methanol, etc. [12]. Conventional extraction methods such as maceration and Soxhlet extraction are widely used to extract compounds from plants. However, they are usually non-selective, time-consuming, and sometimes they degrade heat-sensitive substances [107]. Other modern extraction techniques with novel extraction methods are the ultrasound-assisted extraction, the microwave-assisted extraction, the enzyme-assisted extraction, and the supercritical liquid extraction, which possess certain advantages [103], considering eco-friendly, green technologies for plant extraction.

### 10.1. Maceration

Maceration is the simplest extraction technique in terms of the required equipment. The process can be carried out in an analytical beaker suited for thermolabile compounds. However, because the extraction is carried out at ambient temperature and atmospheric pressure, it usually leads to low efficiency, which is why the technique requires a higher volume of reagents and a longer extraction time [111].

### 10.2. Pre-Treatment

The pre-treatment consists of the defatting of plant material, which is essential for sample preparation. Material can be defatted with chloroform or a chloroform:hexane (1:1) (*v*/*v*) mixture in a Soxhlet apparatus for 48 h [112].

### 10.3. Extraction

The maceration technique is the most common and useful method to isolate saponins from *M. sativa* L. and from other plants [113]. The solvent selection is performed based on the solubility of the biologically active compound (solvent polarity) and the solvent evaporation temperature [114]. The defatted and dried material is immersed in 80% aqueous methanol under reflux for 1 h or three times at room temperature for 24 h [112]. Tava et al. [115] used 80% methanol to extract saponins from *M. arabica*. The highest content of sapogenins (compared with other extraction techniques SE, SFE) in the methanol extract was determined in the roots of *M. sativa*; however, the method has no specificity in relation to the extraction of biologically active compounds [116]. Khaledi et al. [117] used water and butyric acid-free bitter ethanol at a 30:70 ratio for the extraction of biologically active compounds. Amber et al. [118] performed maceration of flavonoids, alkaloids, and saponins.

Chegini et al. [119] used maceration to obtain a biologically active compound from *M. sativa*. Golla et al. [120] extracted peptides from seeds and used salt precipitation with a phosphate buffer against freeze-dried seeds. Ethanol solution is also a good option for extracting proteins [121] and flavonoids [108]. Rodrigues et al. [122] performed maceration with 20 mL of 50% ethanol at 40 °C for 30 min to extract active ingredients from *Medicago* spp. leaves. Sangwan et al. [123] used extraction with ethanol for *T. aestivum* and *Terminalia bellirica* leaves. Hadrich et al. [124] carried out sequential maceration of biologically active compounds from the pea plant with three solvents with increased polarity: ethyl acetate, methanol, and water. Sundaresan et al. [125] carried out the extraction of wheat grass bioactive compounds by using different solvents—water, methanol, ethanol, hexane, and ethyl acetate. Kim et al. [126] used chloroform to extract quinone compounds from the wheat germ. Nair et al. [127] used the maceration approach to extract lecithin from peas (*P. sativum*) and lentils (*Lens culinaris* L.), jackfruit (*Artocarpus heterophyllus* Lam.) and jack bean (*Canavalia ensiformis* (L.) DC.), while ammonium sulfate was used to extract lectins. Narendhirakannan et al. [128] used the Soxhlet apparatus with ethanol as a solvent to extract active compounds from seven traditional medicinal plants easily accessible in India. The aqueous acetone solution was used to extract phenolic compounds [109]. Pure water is also a possible reagent for extracting phenolic species from the flowers of *M. sativa*; the average content of bioactive compound in the methanol extract was 263.5 ± 1.02 mg GAE/100 g of the dry-weight lyophilized extract [129].

### 10.4. Purification, Quantification

Purification is followed by quantification with RP-HPLC-UV to remove sugars and some phenolics. Bialy et al. [130] obtained extract from *Medicago sativa* L. containing glycosides and triterpene compounds from the extract of saponins by chromatographically treating the n-BuOH eluent saturated with water. The peptides were purified from the solution by precipitation of ammonium sulfate and separated on a 5 kDa membrane in the spin column [120]. Nair et al. [127] used dialysis against Tris, PBS, and NaCl gradient for the purification of isolated compounds. Troszynska et al. [109] applied column chromatography with the same mobile phase composition as an extraction reagent to the fractionation and purification of the extract obtained.

### 10.5. Derivatization

Extracted saponins can be derivatized to enhance their solubility [131]. Abbruscato et al. [132] obtained prosapogenins from part of the fractionated solution after alkaline and acid hydrolyses.

### 10.6. Accelerated Solvent Extraction (ASE)

ASE is based on using a solvent above its boiling temperature alongside higher pressure in the reagent cell, which enables higher extraction efficiency, lower reagent use, and shorter process time in comparison to maceration. However, the extraction is performed with expensive equipment. The sample pretreatment is similar to the maceration procedure, but some instrumental and physicochemical parameters (temperature, pressure) can be adjusted and optimized [133]. A smaller particle size, in most cases, leads to higher extraction efficacy [134]. However, the ASE extraction yielded similar results to maceration in the anthocyanin extraction from blue wheat, purple corn, and black rice conducted at optimal conditions [135]. Aqueous ethanol was most effective in extracting phenolic species from whole grain and bran of both soft and hard wheat [136]. What is more, aqueous methanol gives a higher extraction yield in comparison to hexane and acetone when phenolics were isolated from wheat brans [134]. The ASE extraction is more efficient in yield and retains the biological activity better in comparison to hot water maceration (determined according to oxygen radical absorbance capacity, DPPH, and hydroxyl radical scavenging ability) of polysaccharides extracted from *Chimonobambusa quadrangularis* [137]. The ASE extract from flowers of *M. sativa* (70% ethanol) was most efficient in the extraction of compounds regarding the total phenolic content and antioxidant activity [138]. By using ASE, the lipophilic vitamin K1 can be extracted with an n-heptane:ethyl acetate (4:1) solvent from the pea plant, in which the solid phase extraction was performed after purification from other lipophilic substances [139].

### 10.7. Supercritical Fluid Extraction (SFE)

Using a solvent in the supercritical state provides an opportunity to reach a high extraction yield; the fast experimental process gives the highest versatility for the extraction of biologically active compounds which differ in polarity [140]. The most common fluid is carbon dioxide which is non-polar in the supercritical state; by using a co-solvent (ethanol is the most common) with hydrophilic properties, selectivity of the extraction can be obtained [10]. For example, sequential extraction was performed in the selective isolation of nonpolar and polar compounds from triticale cultivars; in the first step, carbon dioxide allowed selective isolation of alkylresorcinols, while the second step involving ethanol as a cosolvent enriched polar lipids in the second fraction [141].

A smaller particle size leads to higher extraction efficiency [110]. According to Krakowska-Sieprawska [142], enzymatic degradation of alfalfa leaves enhances the solvent’s penetration of the material and consequently ensures a higher extraction yield. Using the enzymatic degradation of *M. sativa* leaves resulted in an almost 50% higher yield (quantified by HPLC-MS polyphenols analysis) compared to a nondigested sample [143].

The highest efficiency in terms of the whole group of compounds can be achieved in higher pressure and temperature extraction conditions (300 bar and 80 °C, respectively), while higher specificity can be obtained at lower values of these parameters (200 bar and 40 °C) in the extraction of lupane-type triterpenoids from *Acacia dealbata* bark [144]. In contrast, the settings of 200 bar and 40 °C were sufficient to reach the maximal and individual (specificity) efficiency in the extraction of compounds from *Pterodon* spp. [145]. The highest extraction efficiency and the content of vouacapan diterpenes were reached at 220 bar and 40 °C, while the extract of *Ptedoron* spp. plant obtained at 160 bar and 50 °C had the highest antioxidant activity [146]. The settings composed of 270 bar and 42.6 °C resulted in the highest efficiency during the extraction of compounds from *Trigonella foenum-graecum* L., while lower temperature and pressure enabled the isolation of higher amounts of sterols, vitamin E, and vitamin D [147]. The diosgenin yield from the extraction of *T. foenum-graecum* L. seeds depended particularly on the pressure and carbon dioxide flow [146]. Ge et al. [148] concluded that the extraction of vitamin E from wheat germ with SFE was potentially better than conventional techniques. SFE is popular for extracting biologically active compounds from wheat germ oil [149].

### 10.8. Microwave Assisted Extraction (MAE)

MAE is used in the extraction of biologically active compounds from plants, while the proper selection of temperature, power, and time settings is crucial to retain the biological function of the compounds. The following settings: 70 °C, 300 W, and 10 min in MAE, were the most effective in extracting anthocyanins from blue wheat and purple corn, compared with 50 °C, 1200 W, and 20 min for black rice [135]. A level of 90% of the nominal microwave power, 30 s irradiation time, and 1.5 g sample size were optimal to extract phenolic antioxidants from peanut skins with 30% aqueous ethanol in accordance with the total phenolic content parameter [150]. The MAE technique (methanol as a solvent) was more effective in comparison to maceration in an antioxidant extraction study on wheat bran, taking into consideration the total phenolic compounds, catechin equivalent, total tocopherol content, and Trolox equivalent [151].

## 11. Techniques Applied in the Determination of Silicon in Plants

### 11.1. Direct Techniques

#### 11.1.1. XRF

Dissolution always carries a risk of using a hazardous reagent and a time-consuming utilization procedure; in particular, silicon (Si) may cause trouble by solubilizing from the complex matrix, as a result of which the analysis can be charged with low accuracy [152]. Thus, silicon can be analyzed in the plant material using direct techniques, which do not require silica solubilization. Such analytical techniques do not entail the decomposition of a solid sample (the signal from a sample in the solid phase is measured). An example is the XRF technique (X-ray fluorescence). The measurement of a sample consists of an irradiation process carried out by the primary X-rays in order to subsequently analyze the secondary X-ray spectrum emitted by the sample itself. It is known to be a fast, safe, nondestructive, and potentially more accurate analysis [77]. Reidinger et al. [77] used a portable spectrometer. They showed a detection limit of 140 mg Si/kg. The technique fulfills validation parameters, and a large number of samples (200 per day) can be tested with this technique. Guerra et al. [153] carried out experiments on the determination of the real-time concentrations of nutrient elements (K, Ca, S, Si) in plants, and the results (in g/kg sample) were close in value to the validated comparative method (ICP-OES).

However, the XRF determination revealed some inaccuracies in the air absorption phenomena and evinced a high drift of the spectrometer [154]. For light elements (lighter than 31 in mass units), the measurement has to be performed in a neutral gas environment [155]. Thorne et al. [154] overcame these drawbacks by performing an analysis of silicon in wheat in a helium atmosphere (reducing the first effect, adopted from [156]) and replicating the analysis after 1 h (reducing drift, adopted from [154,157]). Van der Ent et al. [155] used the helium environment and stated that silicon accumulated in the base of the trichomes. According to Deshmukh et al. [158], soybean plants accumulated 2.5 times more Si in leaf tissue in comparison with the control plants grown without Si supplementation. Baranowski et al. [159] performed a speciation analysis in soil and showed that the form of silicon depended on the type of soil and the origin of the samples.

#### 11.1.2. Laser Ablation

Another technique for analysis in the solid phase is LA-ICP-MS. LA uses UV-nanosecond LA to generate an aerosol from the solid surface of a sample. The most critical step of sample preparation consists of the deposition of a sample in the cell [160]. Frick et al. [160] examined the initial digestion. Purification gives the best results in terms of reproducibility and repeatability of determinations of silicon in soil and plant extracts [160]. Fleck et al. [161] prepared samples using a modified Steedman’s wax protocol. In the root of rice, the determined silicon concentration was the highest in the outer cell layers comprising the exodermis and the sclerenchyma; it gradually decreased towards the central cylinder in the endodermis of the rice roots. It was revealed that the rice plant accumulated silicon, especially in the roots, and LA made it possible to determine the silicon concentration in the interior and exterior parts of the roots; the silicon concentration was significantly higher in the interior part of the roots than in the surface [162].

#### 11.1.3. Laser-Induced Breakdown

A high-energy laser (Nd:YAG) beam is focused on a sample, which ablates a tiny part of the material, and plasma is generated. Tripathi et al. used laser-induced breakdown spectroscopy to determine silicon in different parts of the wheat plant. They revealed that leaves had the highest silicon content [163].

#### 11.1.4. Electrothermal Vaporization

The ETV technique transfers elements from the solid phase of a sample to a mass or optical spectrometer (ETV-ICP-MS or ETV-ICP-OES). Masson et al. achieved the limits of detection at the content range of 30 µg Si/g in the plant material. Moreover, cellulose was found to be the ideal support for matrix matching of calibration curves [88].

#### 11.1.5. EDX

SEM-EDX is based on the generation of characteristic X-rays in atoms of a specimen using the incident beam electrons. Soukup et al. [164] used the SEM-EDX technique to detect and measure the silicon content in plant material. In this study, silica was aggregated in the non-lignified parts of the endodermal cell walls [164].

### 11.2. Indirect Techniques

ICP-OES is the most popular analytical technique applied to determine the concentration of silicon in a solution of plant material. Owing to its popularity, availability of the equipment, and relatively low price, the technique is adequately sensitive and insensitive to matrix components, for example, compared to ICP-MS [165]. The silicon quantification is not burdened with spectral interferences. This is the most popular technique in plant research reported in the subject literature.

#### 11.2.1. ICP-OES

ICP-OES is an analytical technique that allows the spontaneous emission of photons from atoms and ions that have been excited in a plasma torch device. Barros et al. [82] reported limits on detection and quantification in the range of 56 and 186 mg Si/kg for the optimized sample preparation protocol, and recoveries were in the range of 91.0–109% of the silicon determination in apple leaves, tomato leaves, white cabbage powder, bush branches, and leaves of CRM materials [82]. The silicon content in plant-certified reference materials is in the range of 0.2–30 g/kg [166]. Method detection limits in the range of 0.24 and 0.024 mg/kg were determined in the matrix solution—HF-H_3_BO_3_ and 2% HNO_3_, respectively—and used to quantify Si in sugar cane leaves (mean Si content was 5522 ± 1410 mg/kg) [167].

Ramírez-Olvera et al. [168] studied the Si concentration in rice plants under conventional and osmotic stress conditions. They showed a decrease in K and Mn in roots after the silicon supplementation and an increase in Fe and Zn in shoots. Quigley et al. [165] reported the limit of detection at r 10 µg Si/L, and the recovery of the silicon determination was higher than 95% with respect to the certified reference material, which was hay. The validated method served to carry out Si determination in more than 800 plant samples, including forbs, legumes, and raminoids [165]. Guntzer et al. [83] tested the solvent for sample preparation and tiron for electrothermal vaporization; lithium metaborate fusion gave lower recoveries. Gu et al. [169] studied the influence of zinc and silicon on rice and stated that the ameliorative mechanism of silicate on excessive zinc toxicity to rice could be attributed to an internal plant response.

#### 11.2.2. ICP-MS

ICP mass spectrometry (ICP-MS) is a powerful tool that makes an analysis of ultra-trace level concentrations possible for most elements in the solution. In this technique, plasma is used to create single positive ions of elements in the gas phase; the ions are separated by their mass-to-charge ratio (*m*/*z*) in a mass analyzer. However, silicon determination encounters many difficulties. A single quadrupole mass analyzer, due to its resolution of about 0.8–0.7 atomic mass units, is not sufficient to distinguish the analyte from spectral interferences- ^14^N_2_, ^12^C^16^O; analyzing the most common silicon isotope 28Si (92% abundance), strong in intensity, is difficult at a trace level concentration [170]. In comparison with other elements, the background of the 28 *m*/*z* line is definitely higher. Mihaylova et al. [171] recommended the cold plasma ionization condition (RF power of 600 W) for ^28^Si quantification in the plant matrix. Additionally, sample matrix (the residue of plant material after digestion and solvents) enhances observed signals for low-mass elements in particular [172], therefore, appropriate calibration methods should be used. The popular approach based on the collision cell (kinetic energy discrimination one) applied to reduce spectral interferences in many cases is insufficient. The high background can be reduced by replacing glass equipment in the spectrometer. Aureli et al. [173] showed reducing *m*/*z* 28 lines by 20% when the glass equipment (nebulizer, spray chamber, torch) was replaced by Teflon devices. Additionally, they used the dynamic reaction cell to reduce spectral interferences and achieve the limits of detection lower than 1 µg/g for most samples. The proper selection of instrumental parameters, such as power on the plasma coil, flows of plasma/collision gases, voltages on cells and lenses, etc., is crucial for obtaining as low a background concentration as possible without a significant loss of the analyte [174].

Using the dynamic reaction cell (to reduce polyatomic interferences generated in plasma) is a very promising solution to drawbacks present in the standard set-up of ICP-MS. Liu and Jiang [175] used ammonia as a collision gas and reported an improvement of one order of magnitude in the signal to background intensities during a silicon analysis in steel. Furthermore, the limit of detection was 0.2 µg Si/L, and the accuracy and repeatability were less than 3% and 5%, respectively. He et al. [96] used this instrumental set-up in their study and showed that more silicon accumulated in the cell wall with hemicellulose and pectin than in the cell wall without pectin or without hemicellulose and pectin.

The lowest detection limit of Si and, more importantly, BEC can be obtained by applying the high-resolution HR ICP-MS technique. Klemens and Heumann [176] performed silicon determination with isotope dilution calibration and showed detection limits at 0.15 µg/g for the HNO_3_ matrix and 0.2 µg/g when HF was also present in the matrix, while the repeatability was in the range of 2–4% for homogeneous biological and clinical samples.

#### 11.2.3. Colorimetric Method

Historically, the standard method for colorimetric determination of silicon is based on the formation of the blue complex compound between silicon and molybdenum blue and the determination of the solution’s absorbance on a UV-VIS spectrometer at 660 nm wavelength. This method can be applied to a plant matrix but will only identify rather high silicon concentrations due to its limited sensitivity. Researchers have noted the tendency towards a lower silicon content than in the control after the Cd stress of rice [98].

Pan et al. [103] showed the Si-defective mutant (Lsi1) of rice did not absorb silicon from the environment, but wild rice plants adsorb this element. Mitani and Ma [177] determined Si concentrations in rice tissue and reported its absorption of up to 10% of dry weight in the plant shoots (rice has a high ability to load Si into the xylem). Liang et al. [178] showed that both active and passive Si-uptake co-occurs in *O. sativa*, *Z. mays*, *H. annuus*, and *B. hispida*. Hodson et al. [179] collated and compared silicon concentrations in many plants (data from 125 studies and species in relation to the Poales family) and showed that, in general, ferns, gymnosperms, and angiosperms accumulated less silicon in shoots than non-vascular plant species and horsetail.

#### 11.2.4. Silicon Speciation

A coupled separation device (liquid chromatography equipment is the most popular) for detecting the sensitivity to silicon compounds enables research on silicon speciation. Chemical speciation deals with the identification and quantification of the distribution of elements in a sample [180]. Silicon can be present in many chemical forms, especially in plant tissues. Analysis of many silicon forms may be hampered by several analytical problems because of differences between the chemical and physical properties of these compounds, most significantly solubility, viscosity, and surface tension of dispersion; the same concentrations of Si in various forms of silicon did not induce the same detector’s response by intensity (OES detection) [181]. The discrepancies among signals may have been caused by the solution transport process, thus, the knowledge of the sample matrix and matrix of standards to the silicon chemical form must be taken into consideration to guarantee reliable results [181]. Ebdon et al. [182] performed polar silicon speciation by reverse-phase HPLC with the ICP-OES axial and radial view in the 1990s and reported that BEC values were similar and were about 0.9 mg/L for the axial and radial analytical mode. The sensitivity of the axial mode was significantly higher for the water matrix of the solution but similar for organics. The limits of detection were 0.1 mg/L to 0.5 mg/L for inorganic Si and hexamethyldisiloxane, respectively [182]. Carter et al. [183] used the size exclusion and reverse phase mode of chromatography coupled with the sector field ICP-MS and showed the limits of detection for silicon compounds at 12–30 µg/L and 0.1–4 µg/L for the PDMS and silanol compounds, respectively.

NMR can be used to obtain information about chemical forms of silicon in plants. The identification is based on the number of peaks and their position in a scale (chemical shift) and comparison with the standards. Si was detected in the xylem of *Oryza sativa* L. in the chemical form of monosilicic acid [177]. Ma [184] expanded research in this area and demonstrated the same silicon form in the root, xylem, and leaf in low-silicon rice mutant (Lsi1) and wild-type rice. However, Park et al. [185] showed three silicon forms in four rice cultivars, namely the homologs Si(OSi)_4_, Si(OH)_1_(OSi)_3,_ and Si(OH)_2_(OSi)_2_, were detected in rice plants. Casey et al. [45] showed two forms: mono and disilicic acid, in a ratio of approximately 7:1, present in wheat (*T. aestivum* L.). Schaller et al. [186] detected tri- and tetra-silicic acid in common reed (*Phragmites australis*).

In addition, Cabrera et al. [187] studied the effect of lignin-silica complexing by characterizing lignin and silica coprecipitates with FTIR and solid-state NMR using soda lignins, such as protobind 1000 and 6000 (plant source: wheat straw), and organosolv lignin (plant source: mix of maple/birch/poplar), reporting interactions between lignin and silica through hydrogen bonds, and reaffirming that lignin induced the substitution pattern changes in the silica surface.

#### 11.2.5. Organic Silicon Compounds in Plants

Direct evidence for the presence of organic derivatives of silicon compounds is difficult to find in the present literature. The adsorption of oleic acid onto silica gel taken from ashed rice hulls is an exergonic reaction with ΔG equal to −23.1 kJ/mol [188]. Furthermore, it should be noted that the derivatives of silicon compounds in plants resulting from covalent interactions (bonds) are not yet known or described. However, Kinrade et al. [189] showed that silicon could create a stable compound with polyols, wherein the compound is five- or six-coordinated and ligands are specifically localized in space. The aqueous catechol solution incites the formation of a Si-catechol complex with a coordination number of six [190]. Kinrade et al. [189] provided evidence in the form of an NMR spectrum for the existence of gluconate derivate of a silicon compound in the dilute solution with a pH similar to the pH of the soil solution. Silicate cross-linking to the cell wall of xyloglucan has also been reported [191].

In accordance with Korndörfer et al. [192], who collected works about organo-silicon compounds in plants, Inanga et al. [193] analyzed the cell wall of rice plants subjected to the silicon solution and demonstrated using IR and UV spectrometry the possible presence of silicon bound to some organic compounds. According to Matychenkov et al. [194], organo-silicon compounds are classified as the soluble fraction of silicon, and these compounds were created by hydrogen bonding. In general, biomacromolecules are involved in the transport and polymerization process of silicon in the cell wall [195,196]. Ishii and Matsunaga [197] isolated an alcohol-insoluble fraction from rice seedlings. Subsequently, a water fraction was obtained by enzymatic degradation of the cell wall. In this water fraction, silicon was found through SEC-ICP-OES to occur as bound to high molecular weight compounds (probably polysaccharides) [197]. Moreover, a relevant role of peptides and proteins in the accumulation, transport, and metabolism of silicon compounds is revealed in the literature. Peptides and amino acids could form polysilicic species through interactions. Sahebi et al. [198] showed a key role of some proteins rich in serine and proline in the absorption and accumulation of silicon in the epidermal root cell walls. Kauss et al. [199] showed that specific proline-rich proteins could significantly enhance the silica deposition in the cell wall. Although several reasons for the presence of organic derivatives of silicon have been suggested, there is still a lack of data on the sequencing of individual compounds from plants using coupled techniques, for example, data from MSn-techniques.

#### 11.2.6. Microscopy (SEM, TEM, AFM, Light Microscopy) Studies of Silicon in Plants

Silicon deposition can be observed by fluorescent microscopy techniques. Shimizu et al. [200] discussed the fluorescent dye of silicon deposition 2-(4-pyridyl)-5-((4-(2-dimethylaminoethylaminocarbamoyl)methoxy)-phenyl)oxazole (PDMPO), whereby silicon can be visualized at a concentration higher than 3.2 mM. Dabney et al. [201] developed a method to employ autofluorescence to study silica size and distribution in plants. The three types of silica bodies or silica body related to mineral structures were examined in both abaxial and adaxial epidermis of *K. macrantha* leaves. Sizes of these species, density, and distribution patterns varied.

Blecher et al. [202] compared three methods (SEM, light microscopy, and Raman imaging) to observe the silicon deposition in *Equisetum hyemale*, *Carex pendula*, and *Miscanthus sinensis*. They discussed the pros and cons of these solutions, although the combination of EDX and vCD (low-voltage-high-contrast) offered the best possibility for identifying the localization of silica insertions [202].

SEM imaging gives an opportunity to visualize silicon. Alvarez et al. [203] showed large amounts, in comparison to the control, of amorphous silica in the flag leaf blades of rice plants treated with soluble silicate and nanosilica with the comparable density of particles from these two sources. Głazowska et al. [204] showed that low silicon availability in the mutant defective in silicon uptake called low-silicon 1 *Brachypodium distachyon* promotes deposition of Si in the amorphous form or bound to cell wall polymers rather than as silicified structures. He et al. [96] showed, using AFM microscopy that silicon is covalently bound to hemicellulose. In roots, stegmata cells containing Si aggregates were positioned on the outer surface of the sclerenchyma bundles, while silicon was undetected in the epidermis, nor was it found in association with the cuticle [99]. Kido et al. [100] showed that Si was localized in the marginal areas of leaf blades of wild-type rice plants. Hodson and Sangster [205] showed that silicon was localized primarily in the endodermis associated with the inner tangential wall. Zexer and Elbaum [206] detected putative silica aggregation loci identified in the roots of *Sorghum bicolor*. An aggregate form of silicon was present in the roots of oat plants (268–366 nm in size), and nanoparticle silicon was also present in the cell walls of leaves [207]. The silica aggregates were lamellar in structure [164]. In contrast, Perry et al. [208] showed that the shape of silica deposited in the lemma of *Phalaris canariensis* L. changed from sheet-like (days 0–11 post-emergence) and globular (days 12–32) to fibrillar (days 33–40) [208,209].

The coupling TEM or SEM with an EDX detector enables the detection of other elements in plant material, whereas the information about these elements is lost after the digestion of solid plant material [205]. For example, the preparation of a sample using digestion with HF excludes the detection of calcium in the sample due to the precipitation of calcium fluoride from the sample. Gu et al. [169] used SEM in a study on rice plants and showed that the highest Si concentration was around the endodermis; the second most abundant sites were around the exodermis and sclerenchyma in roots, while in leaf sheaths, the preferential localization of Si was around the epidermis and sclerenchyma. The impact of iron on the distribution of silicon in the roots and leaves of rice plants was examined. The results displayed the trend for silicon accumulation in the root epidermis and vascular cylinder, glandular trichomes, the epidermis, and the vascular cylinder of leaves of the rice plants grown in a medium with silicon supplementation and absence of iron [210].

#### 11.2.7. Structural Analysis (IR and Raman) of Silicon in Plants

IR spectrometry is a technique to identify compounds owing to very specific absorption of chemical groups of compounds on IR radiation. The presence of silicon can be detected in the range of “the fingerprint region” in a spectrum. Soukup et al. [164] stated that only small differences were observed in the spectra between silicon-supplemented and non-supplemented plants. The differences were visible in regions of the C=O stretching band (1670–1760 cm^−1^), to -OCH_3_ and -OH groups conjugated to aromatic rings (1140–1190 cm^−1^) and to phenolic compounds (1550–1670 cm^−1^) [164]. Bokor et al. [99] showed a broad band in the region 400–490 cm^−1^ and assigned the bond-rocking vibration to Si-O-Si, underlining the amorphous nature of silica. The broad and asymmetrical band around 800 cm^−1^ visible in all three spectra was attributed to symmetric Si-O-Si stretching vibrations [99].

### 11.3. Analytical Techniques for the Separation and Isolation of Biologically Active Compounds

Depending on a plant species, Si source, Si amount, and the plant’s ability to take up Si, the plant Si content can range from 0.1% to 10% (dry weight) [18,211]. The high Si deposition in plant tissues improves their strength and rigidity [14]. On the other hand, phytoliths are found in silica cells in the leaves of Si-accumulating species, such as those belonging to the Poaceae family (rice and wheat), and they vary with the species and age of a plant [212]. In addition, it was reported that most monocots, for example, plants of the Poaceae family such as *T. aestivum* (wheat), *Oryza sativa* (rice), *Lolium perenne* (ryegrass), *Hordeum vulgare* (barley), and *Zea mays* (maize), are high Si accumulators [212,213]; rice accumulates up to 10% Si in shoots [213]. Further, some dicots (Fabaceae family), for instance, fava bean (*Vicia faba*), are found to incorporate Si into their roots [211]. Si doses play a very important role in the accumulation of plant bioactive compounds and the antioxidant capacity, where positive effects of Si are closely related to the accumulation of Si in different plant tissues [214].

**Figure 4:**
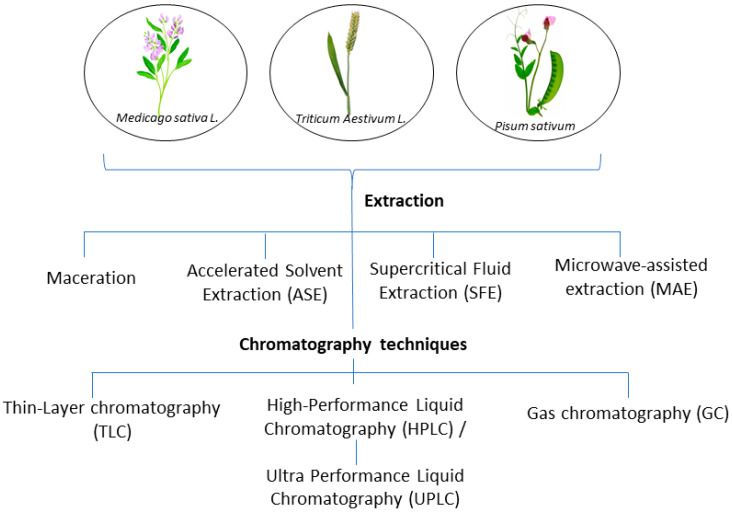
Determination and identification of Si-based biologically active compounds in *Medicago sativa* L., *T. aestivum* L., and *P. sativum* L.

Once plant materials are extracted with different techniques, such as maceration, ASE, SFE, MAE, etc., the extracts are subjected to a separation technique. The chromatographic techniques most frequently applied in the phytochemical analysis are thin-layer chromatography (TLC), high-performance liquid chromatography (HPLC), and gas chromatography (GC); these analytical techniques could also be used in the identification and isolation of individual components from composite mixtures (preparative and micro-preparative scale) [11] (Figure 4).

#### 11.3.1. Thin-Layer Chromatography (TLC)

Thin-layer chromatography (TLC), also called planar chromatography, is a technique for the qualitative and quantitative analysis of organic or inorganic compounds, isolation of individual compounds from multicomponent mixtures, and preparative-scale isolation; it is also a principal separation technique in plant chemistry research [11,215]. A classical thin layer has a thickness of 250 μm, a particle size of 10–12 μm, and allows an analysis of approx. 10 samples in 0.1 to 1000 μL amount at a max. 15 cm development distance simultaneously; thus, the thickness of the layer (stationary phase) and the particle size distribution based on the properties of the analyte are important for the quality and efficiency of separation [215].

For planar chromatography with high-performance layers, detection, and data acquisition, high-performance thin-layer chromatography (HPTLC) could be used [8], offering more rapid separation with thinner layers, smaller particles, and a narrower particle-size distribution [216]. HPLTC uses pre-coated plates coated with a sorbent of particle size 5–7 microns and a layer thickness of 150–200 microns [8]. Additionally, HPTLC appears to be more sensitive and reproducible than classical TLC [216].

After the biologically active compounds present in a sample are separated using preparative TLC or HPTLC, those compounds can be identified by employing GC-MS and NMR techniques [217], LC-MS [218], LA-ICP-MS [219,220], SEM-EDS, and ICP-OES [221].

The advantages of TLC are particularly important in research on plant extracts, which are very complex mixtures, as it allows the identification of known and unknown compounds, as well as the selection of biologically active compounds [11]. With a minimal sample, the sample preparation can be applied for the separation, quantification, and structural elucidation of secondary metabolites from crude plant extracts [216]. Often, such extracts contain polar (e.g., phenols and tannins) and nonpolar (lipids, chlorophylls, and waxes) ballasts, as well as a fraction of active substances very important for phytochemistry [11].

The study by Rodrigues et al. [222] provided evidence that *Oryza sativa* L. (rice–Poaceae family) produces several phenolic compounds along with phytoalexins, and the use of TLC and HPLC led to the observation that silicon enhances the accumulation of diterpenoid phytoalexins in rice, a potential mechanism for blast resistance. Oksana Sytar et al. [223] studied the content of anthocyanins (a subgroup of flavonoids) in grains and sprouts of *T. aestivum* L. (wheat), such as cyanidin 3-O-rutinoside, cyanidin 3-O-glucoside, cyanidin 3-O-galactoside, and cyanidin 3-O-galactopyranosyl-rhamnoside, using the HPTLC technique. These scholars reported that cyanidin was two- to three-fold more abundant in the grains than in the sprouts.

#### 11.3.2. High Performance Liquid Chromatography (HPLC)

High-performance liquid chromatography (HPLC) is a popular, versatile, robust, and widely used technique for the analysis and isolation of bioactive natural products [11,12]. HPLC allows the separation (or partition) between a mobile phase (eluent) and a stationary phase (the column packing); hence, depending on the nature of the stationary phase, the separation process can be (i) based on repeated adsorption–desorption steps, (ii) based on the separation between the mobile and the stationary phase, (iii) the stationary phase is made up of an ionic surface of the opposite charge to that of the sample, and (iv) size exclusion chromatography, where the sample is separated according to the molecular size of its constituents [224]. The mobile phases generally employed are water/acetonitrile or water/methanol, which may be run in the gradient elution mode, or in the isocratic elution mode [11]. Natural products are frequently isolated following the evaluation of a relatively crude extract in order to characterize the biologically active entity, which is often present only as a minor component in the extract; the HPLC resolution is suitable for fast processing on an analytical and preparative scale of multicomponent samples [12]. For the phytochemical analyses, HPLC is used for (i) the isolation and purification of secondary metabolites from a complex mixture present in an extract or its fractions, (ii) metabolomic study or principal component analysis, and (iii) quantitative and qualitative analysis [225].

HPLC can be used with a different detector as HPLC-ICP-MS; this technique coupled to ICP-MS is the most important hyphenated technique for the multi-elemental speciation analysis of biological and environmental samples, which requires liquid samples [226]; and LC coupled with a UV–Vis detector is a powerful tool for the separation and identification of anthraquinones, flavonoids, carotenoids, anthocyanins, or indigoids [227].

On the other hand, ultra-performance liquid chromatography (UPLC) is a modern chromatographic system, which is simply the modification of HPLC [228] rooted in the same basic principle, where the main difference between the UPLC and HPLC is in the particle size of the sorbent of the column, namely UPLC is applied in assays of samples with smaller particles <2 μm in diameter; it also operates at high pressure (6000–15,000 psi) and uses 1.7-μm reverse-phase packing material, while conventional HPLCs use 3–5 μm packing material and are set at different pressure (2000 and 4000 psi) [229]. Even more, to improve the UPLC efficiency, a high temperature is set to help reduce the viscosity of the mobile phase, and if the flow rate is high, mass transfer is increased by increasing the diffusivity of the analytes on the column, thereby reducing the back pressure significantly [230,231]. Thus, UPLC has better resolution, speed, and sensitivity compared with HPLC [228]. The application of UPLC in the phytochemical analysis is similar to that of HPLC, namely the identification and quantification of components in a complex mixture; it is the technique’s principal application, i.e., the identification and detection of all possible metabolites in a mixture, in a crude extract or in fractions [225].

Vega et al. [231] explored the impact of silicon on phenolic compounds found in shoot extracts of *Hordeum vulgare* (barley–Poaceae family) under aluminum stress conditions. They discovered that silicon influenced the production of a particular group of flavonoids known as flavone-glucosides. With the help of HPLC, they identified several flavone-glucosides, including isoorientin-7-O-glucoside (lutonarin), apigenin-pentoxide-hexoside isomers 1 and 2, and isoorientin and isovitexin derivatives containing sinapoyl and feruloyl moieties.

In another study, Troszyńska et al. [109] analyzed the seed coat extracts of *Pisum sativum* (pea–Fabaceae family) to determine the presence of various phenolic acids, such as benzoic (p-OH benzoic acid, protocatechuic acid, vanillic acid) and cinnamic acids and their derivatives (*p*-coumaric acid trans, *p*-coumaric derivatives, ferulic derivatives). They also detected flavones (apigenin-7-glucoside, apigenin-8 C-glucoside, luteolin-7-glucoside) and flavonol glycosides (kaempferol-3-glucoside, quercetin-3-rutinoside, quercetin-3-ramnoside) through HPLC assays, emphasizing the potent antioxidant activity of the analyzed extracts.

Kabir et al. [72], on the other hand, investigated the role of silicon in alleviating cadmium toxicity in *Medicago sativa* L. (alfalfa–Fabaceae family). They analyzed plant metabolites, including glutathione, cysteine, methionine, and proline, via HPLC. These metabolites are crucial in combatting oxidative stress induced by cadmium in alfalfa plants.

**Figure 5:**
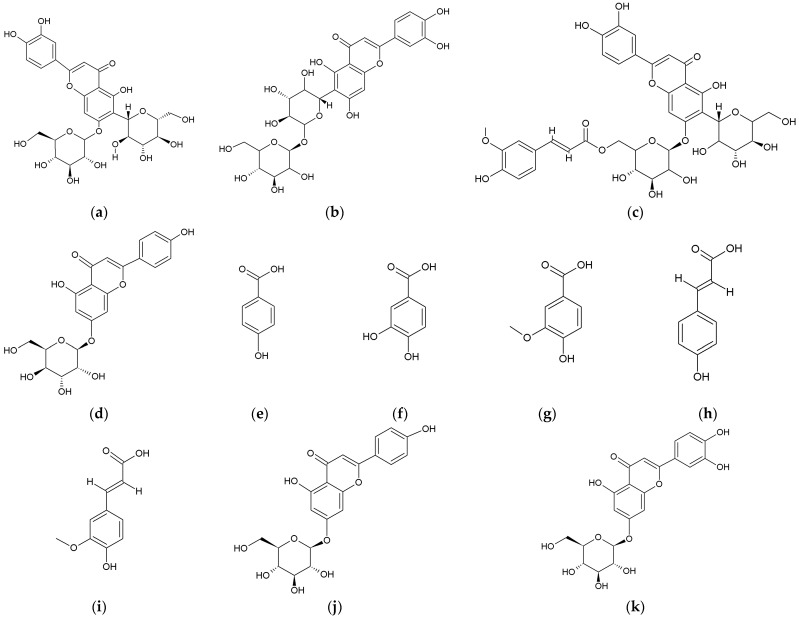

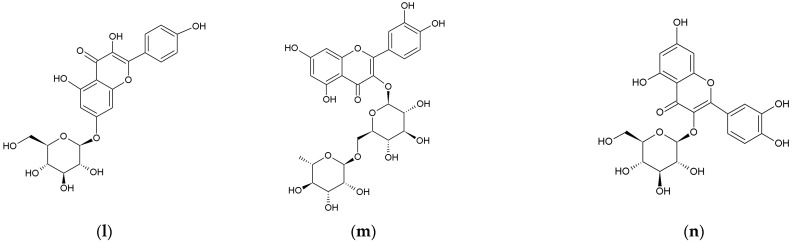
Chemical structures of selected phenolic compounds: (**a**) isoorientin-7-O-glucoside; (**b**) isoorientin 6″-O-glucoside; (**c**) isoorientin 7-O-(6‴-O-(E)-feruloyl)glucoside; (**d**) apigenin-7-O-beta-D-glucopyranoside; (**e**) 4-Hydroxybenzoic acid; (**f**) 3,4-dihydroxybenzoic acid; (**g**) vanillic acid; (**h**) 4-hydroxycinnamic acid; (**i**) ferulic acid; (**j**) apigenin 7-glucoside; (**k**) luteolin-7-glucoside; (**l**) kaempferol 7-O-glucoside; (**m**) quercetin-3-rutinoside; (**n**) quercetin-3-glucoside.

Figure 5 illustrates the chemical structures of selected phenolic compounds, focusing on the flavonoids and phenolic acids described in the literature [72,109,232].

Researchers have suggested the presence of several potential links between silicon and phenol metabolism: (i) silicon could interact with the OH groups of phenols by condensing with Si(OH) in biological systems, (ii) silicon may be involved with lignin–carbohydrate complexes present in cell walls, and (iii) silicon might play a role in signal transduction pathways responsible for inducing lignin production [193,233,234]. These potential mechanisms offer insight into the complex relationship between silicon and the production of secondary metabolites in plants. Silicon has been reported to enhance the production of secondary metabolites derived from the shikimate pathway, which is responsible for the synthesis of aromatic amino acids and phenolic compounds. Studies have suggested that silicon may indirectly modulate this pathway by increasing the activity of key enzymes, such as 3-deoxy-d-arabino-heptulosonate 7-phosphate (DAHP) synthase, shikimate kinase, and chorismate synthase. Enhanced production of secondary metabolites through the shikimate pathway has been associated with improved plant resistance to both biotic and abiotic stresses, as many of these compounds possess antioxidant and antimicrobial properties [5,14,235]. The malonate pathway plays a crucial role in the biosynthesis of various polyketide secondary metabolites, including flavonoids and stilbenes. Silicon may influence the malonate pathway by regulating the expression of genes encoding key enzymes, such as chalcone synthase and stilbene synthase. Additionally, silicon might modulate the activity of these enzymes through post-translational modifications or by affecting substrate availability. As a result, silicon could enhance the production of specific secondary metabolites that contribute to plant defense mechanisms and overall health [5,14,235]. The mevalonate pathway is responsible for the biosynthesis of isoprenoids, a diverse group of secondary metabolites that include terpenoids, carotenoids, and steroids. Silicon has been reported to stimulate the activity of key enzymes in this pathway, such as hydroxymethylglutaryl-CoA reductase (HMGR) and isopentenyl pyrophosphate (IPP) isomerase. The exact mechanism by which silicon affects these enzymes is not yet fully understood, but it may involve changes in gene expression, enzyme activity, or substrate availability. The enhanced production of isoprenoids can lead to increased stress tolerance in plants, as many of these compounds have antioxidant, allelopathic, and signaling properties [5,14,235]. It is worth highlighting the potential role of silicon in modulating the shikimate, malonate, and mevalonate pathways, leading to the enhanced production of secondary metabolites that contribute to plant defense and overall health. Although our understanding of the specific mechanisms by which silicon influences these pathways remains incomplete, our findings contribute to the growing body of evidence suggesting that silicon plays a crucial role in regulating a plant’s secondary metabolism.

#### 11.3.3. Gas Chromatography (GC)

Gas chromatography (GC) is used for assays of volatile and nonpolar (hydrophobe) compounds [11]. Gas chromatography is usually coupled with mass spectrometry (GC-MS), allowing the measurement of the molecular weight of a compound, and once a molecular ion has been identified, it is possible to measure this ion accurately to ascertain the exact number of hydrogens, carbons, oxygens, and any other elements that might be present in the molecule [11]. A conventional GC-MS is composed of an injection port, a detector, and a narrow, coiled column; the mobile phase is often carrier gas, such as helium, nitrogen, or argon; thus, a sample is injected, vaporized, and propelled into a column with the carrier gas [225]. For an analysis of secondary metabolites, volatile and nonpolar ones, GC-MS is a valuable analytical technique, e.g., in assays of stable and volatile alkaloids; nevertheless, flavonoids, isoflavonoids, and hydroxylated alkaloids are converted into derivatives for analysis with GC-MS [225].

Karpagasundar and Kulothungan [236] reported a study on *Physalis minima* (Solanaceae family), where 31 bioactive compounds detected in leaves were evaluated using the GC-MS technique; it was concluded that the prevailing compounds were heneicosanoic acid, bicyclo [4.1.0] hepta-2, 4-dien, octadecanoic acid (CAS), stearic acid, and octadeca-9, 12- dienoic acid. In addition, some studies on plants secondary metabolites analyzed using GC-MS were reported in *Origanum vulgare* L. (arvacrol, thymol, γ-terpinene and linalyl acetate), *Ziziphora tenuior* L. (monoterpenes: pulegone, p-menth-3-en-8-ol, isomenthone, and 8-hydroxymenthone), *Clerodendrum viscosum* (steroids, triterpenoids, alkaloids, saponins, flavonoids, and tannins) and in *Zingiber roseum* (terpenoids: α-pinene, β-pinene, p-cymene, limonene, β-cubebene, transcaryophyllene, terpinen-4-ol, α-terpeneol, epi-cubebol, caryophyllene oxide, and verticiole) [225]. However, no identification studies of silica-based bioactive compounds in *Medicago sativa* L., *T. aestivum* L., and *P. sativum* L. have been reported to use the GC-MS technique.

## 12. Antimicrobial Properties of *Medicago* spp., *P. sativum*, and *Triticum* spp.

Medicinal plants, owing to the rich composition of biologically active compounds, are currently considered to be a promising source of substances with a broad range of antiseptic activity. One such popular plant is *Medicago* spp., commonly known as lucerne or alfalfa, which synthesizes many substances possessing biological properties, such as isoflavones, saponins, coumarins, tannins, terpenoids, naphthoquinones, and alkaloids [112,113,237]. Many researchers indicate the high antibacterial potential of extracts obtained from this plant [117,237]. Khaledi et al. [117] indicate that the extract obtained with the maceration of this plant inhibited the growth of *Enterococcus faecalis* at a concentration of 512 µg/mL and effectively killed this strain of bacteria at a concentration of 1024 µg/mL. The authors suggest that the content of phenolic compounds in the raw material may be responsible for this effect.

In addition, Chavan et al. [237] showed that the alfalfa leaf methanol extract exhibits the minimum inhibitory concentration (MIC) at 37 μg/mL, 12.03 μg/mL, and 111 μg/mL against *Escherichia coli*, *Pseudomonas aeruginosa*, and *Staphylococcus aureus*, respectively. Avato et al. [131] investigated the antimicrobial properties of extracted saponins from roots and aerial parts of alfalfa. The results confirmed the ability of the plant extract to inhibit the development of *Bacillus subtilis*, *Bacillus cereus*, *E. feacalis*, *S. aureus*, and *Saccharomyces cerevisiae*. The authors conclude that the antimicrobial activity of *M. sativa* is related to the content of medicagenic acid. Chegini et al. [119] demonstrated the antibacterial effect of alfalfa root extract on the common bacteria in bronchitis and sinusitis. The results showed that the MIC value of the tested extract was 125 mg/mL against *Haemophilus influenza*, *Streptococcus pneumoniae*, and *Moraxella catarrhalis*. However, it was noted that the extract did not affect *S. aureus*. Rodrigues et al. [122] examined water-alcohol extracts obtained from seven *Medicago* species for their antimicrobial activity. The results of the research allowed the researchers to establish the lowest values of the minimum inhibitory concentrations of extracts at 31.3 µg/mL against *K. pneumoniae*, *S. aureus*, and *Staphylococcus epidermidis*, and 125 µg/mL against *E. coli*.

On the other hand, the extracts obtained from any of the studied *Medicago* species were unable to inhibit the growth of *P. aeruginosa* and *Candida albicans*. Antifungal properties of *M. sativa* were reported by Sadowska et al. [112]. They used *Medicago*-derived saponin fractions to counteract the development of *C. albicans*—one of the most common fungal pathogens of animals and humans. The research results showed that the tested phytochemicals exhibit a direct fungicidal/fungistatic activity. The saponin fraction of *M. sativa* proved to be able to inhibit *C. albicans* germ tube formation and eradicate the mature Candida biofilm. In turn, Abbruscato et al. [132] investigated the antifungal properties of saponin mixtures from alfalfa tops and roots against *Pyricularia oryzae*, a fungus that causes rice blight. They confirm that the best antifungal effects on different cultivars of rice are exhibited by a prosapogenin mixture from *M. sativa* tops. Saniewska et al. [238] compared the antifungal activity of saponins isolated from the root and ground parts of *M. sativa*. The results show that saponins obtained from the roots of the plant evince better fungicidal properties. The saponins isolated from *M. sativa* roots at the concentration of 0.1% could totally inhibit the linear growth of *Phoma narcissi*.

Moreover, the linear growth of *Botrytis cinerea* was limited to about 74%, *Botrytis tulipae* to about 68%, *Alternaria zinniae* to about 67%, *Rhizoctonia solani* to about 74%, and *Phoma poolensis* to about 38%. It was revealed that saponins obtained from alfalfa could also be effective against phytoparasites. The activity of alfalfa against parasites has also been confirmed. D’Addabbo et al. [239] studied five different *Medicago* species to determine their nematicidal properties. The application of saponin extract solutions at concentrations of 1000 μg/mL after 24 h of the treatment led to almost complete mortality of nematodes in the case of the three tested *Medicago* species. Therefore, it can be concluded that extracts obtained from *Medicago* spp. may represent an alternative to chemical plant protection products. Further, some alfalfa proteins can exhibit antibacterial properties. Another interesting plant with a wide spectrum of antiseptic properties is *P. sativum* (garden pea). Saeed and Tariq [240] investigated the antibacterial activity of juice obtained from seeds and skin of *P. sativum* in relation to 56 isolates belonging to 11 species of Gram-negative *bacilli*. The results demonstrate good antibacterial properties of both seeds and skin of *P. sativum* juices, with average inhibition zones of 16.30 ± 2.02 mm and 16.39 ± 3.16 mm, respectively. Hadrich et al. [124] investigated the antibacterial and antifungal properties of extracts obtained by the maceration of pea skin. The best properties were reported for the ethyl acetate extract, while the water extract did not exhibit antibacterial properties. The inhibition zone diameter ranged from 13.0 ± 1.0 mm for *Aspergillus niger* to 19.0 ± 1.0 mm for *E. coli*. Nair et al. [127] examined the antibacterial properties of lectin extracts obtained from *P. sativum*. They showed good properties (MIC value was 1 mg/mL) of the tested extracts in relation to *S. aureus*, *B. subtilis*, *E. coli*, and *P. aeruginosa*. However, the study did not show the killing effect of the examined extracts on the tested bacteria strains.

In turn, Wang et al. [241] investigated the antifungal properties of protein isolated from pea seeds with a molecular mass of 11 kDa and a lysine-rich N-terminal sequence. The isolated protein exhibits good antifungal properties against *Physalospora piricola*, *Fusarium oxysporum*, and *Mycosphaerella arachidicola*. Ye and Ng [242] isolated another protein from *P. sativum* with antifungal properties with a novel N-terminal sequence and a molecular mass of 31 kDa. The protein, designated pisumin, shows good antifungal properties against *Pleurotus ostreatus* and *Coprinus comatus* and much weaker action against *R. solani* and *F. oxysporum*. Another interesting study on the antimicrobial action of *P. sativum* compounds was conducted by Rehman and Khanum [243]. They isolated and characterized the antimicrobial peptides from the seed/pod of *P. sativum* against *Micrococcus luteus*, *S. typhi*, *S. aureus*, *S. epidermidis*, *Klebsiella pneumoniae*, *E. coli*, *P. aeruginosa*, *Proteus vulgaris*, and *Pasterurella multocida*. Observations of the inhibition zones and MIC revealed that the two active peptides from the pod, i.e., P7 (~10 kDa), P8 (~11 kDa), and from the seed, i.e., S4 (~19 kDa), S5 (~22 kDa) showed good antibacterial properties, and the most sensitive strain was *S. aureus*. The research on the antimicrobial properties of pea peptides was also conducted by Golla et al. [120], who showed that germinated seeds had the potential to accumulate peptides with antimicrobial activity. The peptides isolated from peas were effective against *E. coli*, *P. aeruginosa*, and *S. aureus*. Moreover, the extracts from *P. sativum* germinated seeds showed the highest inhibition activity among all extracts obtained in this study. The inhibition zone size obtained in this experiment was 8.58 ± 0.03 mm in relation to *E. coli*, 9.35 ± 0.05 mm in relation to *P. aeruginosa*, and 22.16 ± 0.04 mm in relation to *S. aureus*. Erecevit and Kırbağ [244] investigated the antimicrobial properties of fatty acids, vitamins, and flavonoids extracted from peas against *E. coli*, *B. megaterium*, *K. pneumoniae*, *S. aureus*, *C. glabrata*, *C. albicans*, *Epidermophyton* sp., and *Trichophyton* sp. While the analyzed fatty acids show the best antimicrobial effects, the flavonoids did not inhibit the growth of any of the tested strains except *B. megaterium*.

*T. aestivum*, known as common wheat, also deserves attention owing to its rich composition of biologically active substances. As early as 1972, the antibacterial activity of peptides isolated from this plant against *Xanthomonas campestris*, *Pseudomonas solanacearum*, and *Corynebacterium michiganense* was confirmed [245]. Twenty-four years later, Caruso et al. [246] purified and characterized other proteins from the wheat kernel which exhibited antifungal activity against *B. cinerea*, *Fusarium culmorum*, and *Fusarium graminearum*. In 1988, another study demonstrated the antifungal potential of chitinases isolated from embryos of wheat [247]. Recent studies conducted by Narendhirakannan et al. [128] show that ethanolic and aqueous extracts of *T. aestivum* inhibit the growth of *E. coli*, *K. pneumoniae*, *E. faecalis*, *S. aureus*, and *P. aeruginosa* at a concentration level of 12.5 µg/mL. In addition, Pagnussatt et al. [121] proved the antifungal activity of wheat crude protein extracts against *F. graminearum*.

Saha et al. [248] investigated the antimicrobial potential of the methanol and ethanol extracts obtained from leaf, seed, awn, stem, root, and whole plants of two wheat varieties (Kheri and Pavon76). The results indicated that the methanol and ethanol extracts of Pavon76 seed and awn, as well as the whole plant extract, resulted in the highest diameter of inhibition zones in relation to *E. coli* and *S. aureus* in comparison to Kheri extracts. With respect to the root extract, the methanol extract of both varieties turned out to be more effective in relation to the tested strains than the ethanol extract. The authors attribute the antibacterial properties of the tested extracts to the presence of phytochemicals in wheat.

Rajpurohit et al. [249] tested the antimicrobial activity of wheatgrass extract obtained with the cold extract method against some Gram-positive bacteria strains. The MIC values were found to be 1.25% of the extract against *Lactobacillus* spp. and 5% against *S. mutans*. Sundaresan et al. [125] evaluated the antimicrobial activity of wheat grass extracts obtained using five different solvents (water, hexane, methanol, ethanol, and ethyl acetate). The results reveal that all the tested extracts exhibit antibacterial potential against seven food-borne pathogens. Among the investigated extracts, the hexane extracts from seven-day-old wheat grass exhibit the highest antibacterial action, especially against *Listeria monocytogenes* and *Yersinia enterocolitica*. The study conducted by Sangwan et al. [123] assessed the antimicrobial effects of the simultaneous application of 10% *T. aestivium* and 5% *Triticum bellirica* extracts against two fungal and eight bacterial strains tested in the agar cup plate method. The best results were obtained for the aqueous extracts against *M. luteus*, *E. coli*, *P. vulgaris*, *P. aeruginosa*, and *A. niger*, where the inhibition zone diameter was in the range of 21–23 mm. Furthermore, the antimicrobial activity was also satisfactory with respect to the other strains (inhibition zone diameter −16–18 mm). Schalchli et al. [250] evaluated the allelopathic effect of root exudate extracts from wheat on *Gaeumannomyces graminis* var. *tritici* growth. The results established the MIC value for the most effective extract variant at 0.36 mg/mL.

In turn, Amber et al. [118] investigated the antimicrobial potential of phytochemicals (alkaloids, flavonoids, and saponins) and crude methanolic extracts of wheat against *S. aureus*, *K. pneumonia*, and *E. coli*. The MIC values for all the tested strains ranged between 25–50 mg/mL. Sharma et al. [251] determined the antiseptic potential of anthocyanins extracted from three colored wheat varieties of *T. aestivum* viz., as well as uncolored white wheat. The highest antimicrobial activity was noted for the black flour extract, which has the highest content of anthocyanin. The determined minimum microbicidal concentration of this variant against *E. coli*, *C. albicans*, *S. aureus*, and *P. aeruginosa* was established at 200, 200, 100, and 150 mg/mL, respectively. In addition, black wheatgrass juice extracts were also the most effective among all of the tested wheatgrass juice extracts and showed an MIC value in the range of 100–150 mg/mL in relation to all pathogens. Kim et al. [126] showed that the extract of 2,6-dimethoxy-1,4-benzoquinone included in the wheat germ strongly inhibits *S. aureus* and *B. cereus* growth. The Rajoria research team [252] undertook to identify the major bioactive compound that exhibited antimicrobial properties in various organic extracts of *T. aestivum* L. grass. All the extracts obtained revealed the qualitative presence of the most important phytochemicals, such as steroids and cardiac glycosides, tannins, alkaloids, flavonoids, and carbohydrates.

Moreover, the conducted chromatographic analyses revealed that the presence of bioactive compounds in the extracts, e.g., chlorogenic acid, rutin, chlorogenic acid, and tocopherol, were responsible for the maximum noted antimicrobial activity of wheat grass against *S. aureus*, *Salmonella typhi*, and *Vibrio cholerae*. However, growth inhibition was not observed in the case of *Flavobacterium* sp., *E. coli*, *P. stringii*, *B. subtilis*, and *S. faecalis*. Despite this, the authors conclude that the plant submitted to their research, containing many of the medicinally important bioactive compounds, has great potential to be used in medicine for the treatment of diseases caused by pathogenic bacteria.

Besides traditional techniques for identifying plant biologically active compounds with antimicrobial activity, more innovative tools are becoming available. One such interesting method is thin-layer chromatography-direct bioautography (TLC-DB). This technique combines the separation of biologically active compounds on an adsorbent layer with direct biological tests. For analysis, a TLC plate is immersed in the microbial suspension and incubated in appropriate conditions. Then, after the separation of the active compounds, the inhibition zones of microbial growth can be observed on the TLC plate. Therefore, rapid detection of antibacterial compounds in complex plant extracts is possible in both screening and semi-quantitative tests.

Moreover, it is not only the classic TLC technique but also high-performance thin-layer chromatography (HPTLC), planar electro-chromatography (PEC), and overpressured-layer chromatography (OPLC) that can be easily combined with bioautography [253]. After separation and the evaluation of antimicrobial properties, individual compounds can be directly identified on the plate using spectrometric methods. A technique often used for this purpose is matrix-assisted laser desorption/ionization mass spectrometry (MALDI MS). TLC/MALDI enables the analysis of complex organic mixtures [253,254]. Previous reports indicate that this approach is effective in the identification of active compounds of plant origin, such as lipids or flavonoids [255,256]. The use of “direct analysis in real-time” (DART) in conjunction with TLC also seems to be an interesting approach. This method employs the principles of Penning ionization, in which excited gas atoms interact with air molecules, resulting in the production of secondary ions able to desorb/ionize the compounds present on the surface of the tested plate [254]. The simultaneous application of TLC with derivatization, bioautography, and DART-MS was successfully applied for the analysis of plant-origin substances by Bañuelos-Hernández et al. [257].

Besides their well-known antibacterial properties, plant extracts from *Medicago* spp., *P. sativum*, and *Triticum* spp. can also counteract biofilm formation, making them an important area of focus in this chapter. Biofilms, which are complex communities of microorganisms embedded within a self-produced extracellular matrix, can be challenging to treat due to their increased resistance to antimicrobial agents. Consequently, the discovery of plant extracts’ antibiofilm properties could lead to novel therapeutic approaches against biofilm-related infections.

Abouzeid et al. [258] explored the antibiofilm properties of alfalfa exudate and its identified components. The findings suggested that the primary active compounds influencing biofilm formation are canavanine and hyperoside. These compounds reduced the number of *E. coli* isolates with a moderate and strong biofilm-forming potential from 68.42% to 31.58% and 21.05%, respectively. Moreover, research conducted by Chamachar et al. [259] demonstrated that alfalfa extracts have the potential to inhibit *E. coli* biofilm formation by decreasing the expression of the rcsA and papC genes. Another group of active compounds with antibiofilm properties found in *Medicago* spp. are saponins, which possess significant antifungal properties. Sadowska et al. [112] showed that the saponin fraction of *M. sativa* was capable of inhibiting *Candida albicans* germ tube formation and eradicating mature Candida biofilms. In the case of Candida biofilm formation, Psd1 protein isolated from peas [260] and lectins isolated from wheat [261] also proved to be effective. It was demonstrated that lectins could inhibit Candida biofilm formation at a concentration of 50 µg/mL with an efficiency of up to 44%. González-Ortiz et al. [262] also investigated the effect of wheat on biofilm formation. In their study, a 0.5% concentration of soluble wheat-bran extract exhibited antibiofilm potential against *Staphylococcus aureus*.

## 13. The Effect of *Medicago* spp., *P. sativum*, and *Triticum* spp. on Eukaryotic Cells

**Figure 6:**
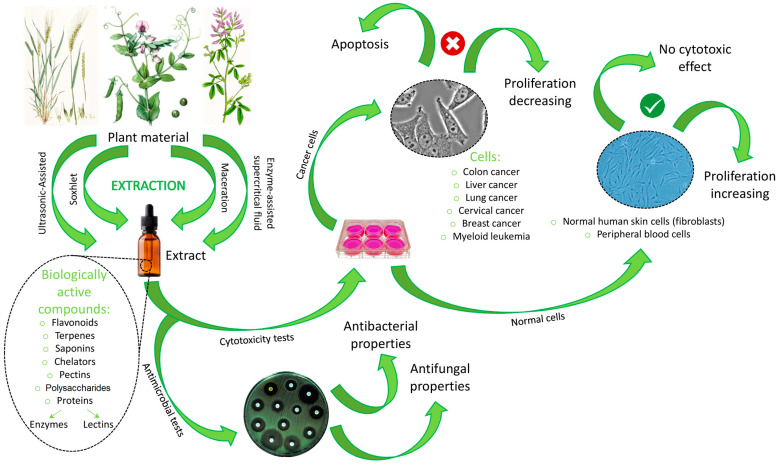
The effect of *Medicago* spp., *P. sativum*, and *Triticum* spp. on eukaryotic cells.

The bioactive compounds present in plants can exhibit cytotoxic properties that have a dual nature in terms of their impact. While their cytotoxic effects on normal, healthy cells may pose limitations to potential applications of plant extracts, the same toxic effects on cancerous or abnormal cells can be highly beneficial. These cytotoxic properties can be harnessed and integrated into existing anti-cancer treatment strategies, potentially improving their effectiveness and offering alternative therapeutic options (Figure 6). This underscores the importance of studying and understanding the cytotoxicity of plant extracts and their active components to maximize their potential in the development of novel anti-cancer treatments.

### 13.1. The Antitumor Activity of Medicago spp., P. sativum, and Triticum spp.

Many studies indicate that extracts and bioactive compounds present in *Medicago* exhibit cytotoxic effects, especially on tumor cells. Gatouillat et al. [263] proved that extract obtained from *M. sativa* leaf could induce apoptosis of sensitive and multidrug-resistant tumor cells. Furthermore, it was determined that terpene derivatives and flavonoids present in the extract were responsible for this effect. The cytotoxic properties were confirmed for chrysoeriol, tricin, (-)-medicarpin, (-)-melilotocarpan E, and millepurpan in relation to the mouse leukemia P388 cell line and its doxorubicin-resistant counterpart (P388/DOX). Further research confirmed that two isoflavonoids: millepurpan and medicarpin, isolated from alfalfa leaves, show a cytotoxic effect on these cell lines. The application of millepurpan and medicarpin leads to the apoptosis of both sensitive and resistant P388 cells. The estimated IC50 values were 54 µM for P388 and 69 µM for P388/DOX after the application of millepurpan and about 90 µM for P388 as well as for P388/DOX cells after the application of medicarpin [264].

Another group of bioactive substances derived from *Medicago* plants that demonstrate antitumor properties are saponins. Research has revealed that mixtures of saponins from various alfalfa species can effectively inhibit the growth of breast cancer (MCF-7) and cervical cancer (HeLa) cell lines [265]. Furthermore, studies conducted on the same cancer cell lines have indicated that a trypsin inhibitor (a low molecular weight protein) sourced from *Medicago* plants could also diminish their clonogenic survival [266]. This evidence underscores the potential value of these biologically active compounds in the development of novel therapeutic strategies for combating different types of cancer.

The active compounds present in *P. sativum* may also exhibit cytotoxic properties. Studies conducted by El-Feky et al. [267] show that the ethyl acetate fraction obtained from peels of *P. sativum* shows a high cytotoxic activity in relation to human breast carcinoma MCF-7 cell line at 73.6% and low cytotoxic action in relation to the colon carcinoma HCT-116 cell line at 21% efficacy. On the other hand, n-hexane extract activity was established at 26.1% and 28.3% for the MCF-7 and HCT-116 cell lines, respectively. More detailed studies allowed researchers to determine the IC50 value of the most effective extracts, which was calculated as 73.4 on the MCF-7 cell line. Furthermore, the antitumor activity of compounds isolated from peas was determined. The most effective compound in relation to the MCF-7 cell line turned out to be apigenin. In addition, this compound was not toxic to normal human skin cell lines. Quercetin turned out to be the least active in relation to the HCT-116 cell line. In another study, it was found that aqueous extracts from the seed coat of dark-colored varieties of *P. sativum* exerted concentration-dependent toxic abilities on human breast carcinoma MDA-MB-453, human colon adenocarcinoma LS174, human lung carcinoma A594, and myelogenous leukemia K562 malignant cell lines. Moreover, the correlation analysis demonstrated that the intensities of cytotoxic properties of the extracts strongly correlated with the concentration of luteolin and epigallocatechin in the extracts. It turned out that the tested extracts were probably able to activate not only caspase-3-dependent apoptosis but also other cell death modalities [268]. 

The activity against HepG-2 cancer cell lines is also exerted by the sulfated alkaline soluble and insoluble extracts from pea peel. In addition, the sulfated acidic insoluble, neutral soluble, and alkaline soluble extracts from the pea peel exhibit activity in relation to MCF-7 cancer cell lines. Their effectiveness was 55.8% and 52.8% (HepG-2), as well as 73.4%, 72.4%, and 72.8% (MCF-7), respectively. For comparison, the effectiveness of Doxorubicin^®^ was 52.6% and 72% for HepG-2 and MCF-7 cells, respectively [269]. It was also proved that the purified asparaginase extracted from peas exhibited a toxic effect on the L20B tumor cell line. A Neutral Red assay was used in this study, which is a cell survival/viability test based on the binding of dye by viable cells. The results indicated that the tested plant-derived enzyme had a significant cytotoxicity effect on tumor cells in the concentration range from 150 µg/mL to 1.17 µg/mL. Moreover, the inhibition of the growth of the L20B cell line was gradually increasing with the increase in the used asparaginase concentration [270]. Another promising compound derived from peas with anti-cancer properties are lectins. El-Aassar et al. [271] demonstrated the cytotoxic properties of lectins extracted and purified from *P. sativum* seeds in relation to hepatocellular carcinoma HepG2 cells. The application of the recommended lectin dose (5 mg/100 μL) results in a decrease in the HepG2 cell proliferation rate by 60.77%. The cytotoxic effect of pea lectins on neoplastic cells was also confirmed by Patel [272]. Some protease inhibitors from peas can also exhibit anti-cancer properties. Clemente et al. [273] reported a significant and dose-dependent decrease in the proliferation of human colorectal adenocarcinoma HT29 cells after the application of the Bowman-Birk trypsin-chymotrypsin inhibitor from peas. The rTI1B had the highest cytotoxic activity in this study (IC50 = 46 µM).

*Triticum* spp. was also found as a potential source of anti-cancer substances. Prolamins obtained from *T. aestivum* spp. *spelta* were found to possess cytotoxic properties against human colon cancer Caco-2/TC7 cells and to agglutinate human myelogenous leukemia K562(S) cells [274]. Barisone et al. [211] investigated the anti-tumor properties of fermented extracts of wheat sprouts (FWGE) and the 10–200 kDa protein fraction (FWGP) obtained from this extract. The lymphomacidal activity of FWGE against NHL cell lines was determined by the evaluation of apoptosis and cell cycle. The determined IC50 was 120, 250, and 275 µg/mL in relation to Jurkat, Ramos, and Raji cell lines, respectively. In addition, the fermented extract was much less toxic against normal human primary B cells (IC50 = 582 µg/mL). However, the results showed that the IC50 value for FWGP was lower than in the case of FWGE. The protein fraction showed significant cytotoxicity to malignant NHL cell lines. Moreover, the cytotoxic action in relation to lung carcinoma (H1650, A549) and hepatic carcinoma (HepG2) cell lines was also shown. The protein extract was only ineffective against breast cancer (MCF-7) cells. Another study assessed the antiproliferative potential of chloroform extract from the dried shoots of *T. aestivum*. The tested extract at the concentration of 250 μg/mL was able to inhibit the growth of 87.23% of the HepG2 human hepatocellular carcinoma cancer cell line [275]. The crude aqueous extract from *T. aestivum* leaves was also tested against the HeLa cell line. The antiproliferative potential of the extract was assayed using the MTT method. The extract exhibited dose-dependent cytotoxic action against the cancer cell line, and, at the same time, it did not exhibit toxicity to the normal VERO cell line [276]. Later studies by the same author confirmed the cytotoxic effect of this extract against the colorectal carcinoma HCT-15 cell line. The application of the MTT method proved the antiproliferative properties of the tested extract, and the IC50 value was estimated at 258.8 μg/mL [277].

### 13.2. The Influence of Medicago spp., P. sativum, and Triticum spp. on Normal Eukaryotic Cells

The widespread application of plant extracts in medicine requires them to fulfill two fundamental criteria. First, they must exhibit cytotoxic properties against cancer cells, and second, they should not have any negative effects on normal, healthy cells. Consequently, it is crucial to study the impact of plant extracts on normal cell lines.

Research on the HaCaT and HFF-1 skin cell lines revealed that hydro-alcoholic extracts from seven different alfalfa species exhibited low cytotoxicity [122]. Additionally, saponin-rich fractions derived from *Medicago sativa* did not demonstrate significant cytotoxic effects on the mouse fibroblast cell line L929 [112]. Another investigation showed that alfalfa leaf extracts obtained using enzyme-assisted supercritical fluid extraction did not reduce the viability of the L929 cell lines up to 0.5 mg/mL, with an IC50 value of 1.36 mg/mL [142]. Furthermore, it was found that plant extracts could also positively influence human cells. *Medicago sativa* extracts obtained through ultrasound-assisted extraction increased the metabolism and proliferation of keratinocyte and fibroblast cell lines. This effect is likely attributed to the high antioxidant activity of these extracts, suggesting their potential use in the cosmetics industry [278]. Additionally, apigenin derived from *Pisum sativum* demonstrated no toxic activity against normal human skin cell lines [267].

In another study, researchers investigated the effects of lectin isolated from peas at a concentration of 5 mg/100 μL, which demonstrated a toxic impact on tumor cells while not causing any adverse effects on peripheral blood mononuclear cells (PBMCs). Interestingly, the study also revealed that using lower concentrations of lectins actually promoted increased proliferation of these cells [271]. Further research by Clemente et al. [279] showed that pea proteins did not hinder the growth of normal, non-malignant colonic fibroblast CCD-18Co cells yet displayed toxic effects on cancer cell lines.

Similarly, the crude aqueous extract derived from *Triticum aestivum* did not exhibit any toxic potential toward the normal VERO cell line. In contrast, it displayed cytotoxic effects on cancer cells [276]. Subsequent studies by the same author confirmed that the *Triticum aestivum* aqueous extract did not exhibit significant toxic properties against fibroblasts (3T6 cell line) at concentrations ranging from 4 to 10 mg/mL. However, the extract did show notable cytotoxicity at a concentration of 2 mg/mL. Remarkably, this concentration also demonstrated greater efficacy in wound healing [280].

## 14. Conclusions

This review shows that silicon has been recognized as one of the most beneficial mineral elements for plants, performing an array of functions, for instance, fortifying plants’ tolerance to abiotic and biotic stresses. Hence, the action of silicon in three plants belonging to the Fabaceae (especially *Pisum sativum* L. and *Medicago sativa* L.) and Poaceae (particularly *Triticum aestivum* L.) families regarding their physiology, strength, and utility for phytoremediation activities has been described in detail. Moreover, the methods of silicon determination and speciation are presented in this paper. Additionally, the review covers current analytical techniques, instruments, and methodologies used to investigate the influence of silicon on plant development. Finally, the manuscript focuses on questions of the isolation and characterization of bioactive compounds in plant material with antimicrobial properties and cytotoxic effects. This review highlights the potential role of silicon in modulating the biochemical and metabolic pathways, leading to the enhanced production of secondary metabolites that contribute to plant defense and overall human health. Although our understanding of the specific mechanisms by which silicon influences these pathways remains incomplete, the literature findings contribute to the growing body of evidence suggesting that silicon plays a crucial role in regulating a plant’s secondary metabolism.

## Figures and Tables

**Table 1:** The role of Si in abiotic and abiotic stress mitigation in *T. aestivum* L., *Medicago sativa* L., *P. sativum* L. [39].

Stress	Example	Plant Species	Role of Si
Abiotic stress			
Drought	-	*T. aestivum* L.	Improves the water relations of wheat leaves, photosynthetic gas exchange, and carboxylase activities in field drought conditions.
Salinity	-	*T. aestivum* L.	Improves the defense by antioxidant enzymes
Salinity	-	*M. sativa* L.	Regulates the K^+^/Na^+^ homeostasis and the leaf water balance, also improving antioxidant enzymes’ performance in leaves.
Heavy metals	Cd	*P. sativum* L.	Accumulation of glyoxalase; it enhances the uptake of macronutrients and micronutrients in shoots and roots
		*M. sativa* L.	Cd binding in both roots and shoots
Biotic stress			
Fungal, bacterial, virus, and herbivores diseases	Powdery mildew (*Blumeria graminis*)	*T. aestivum* L.	Causes a higher concentration of elicitors

**Table 2:** Plant species studied with Si extraction and main techniques of analysis.

Plant Species	Sample Preparation	Techniques	Reference
apple, corn, peach, pepper, watermelon, and bluegrass ground leaves	200 mg of plant tissue with 10 mL of 1 M HCl + 20 mL of 2.3 M HF for 15 h	ICP analysis	[87]
cherry laurel, potato, alfalfa, carnation sunflower, barley grain, grass, French bean, bokashi, oil palm leaf	Plant material at pyrolysis at 420 °C was used	ETV-ICP-OES	[88]
rice (*O. sativa* L.)	Plant tissue added to 2 mL of 30% H_2_O_2_ and 4 mL of 50% NaOH in the oven (95 °C) for 4 h. After, was added 1 mL of 5 mM ammonium fluoride (NH_4_F)	OID MBC using a UV visible spectrophotometer	[89,90,91]
wheat (*T. aestivum* L.)			[90,91]
plant material (29 samples)	30 mg of pulverized plant material was incubated for 4 h in 0.1 M Na_2_CO_3_ at 80 °C. Afterward, 10 mL of the extract was filtered	NIRS	[86]
sugar maple (*Acer saccharum* Marsh.), American beech (*Fagus grandifolia* Erhr.) yellow birch (*Betula alleghaniensis* Britt.)	100 mg of dried tissue was digested in 40 mL of 0.5 M NaOH at 85 °C for 4 h. 30 mg of dry tissue was digested in 40 mL of 0.1 M Na_2_CO_3_ at 85 °C for 4 h	DSi colorimetrically, using the molybdate-blue method	[84,92]
*C. epigejos* and *P. australis*	First digestion: 100 mg plant material digested in a mixture of 4 mL distilled water, 5 mL nitric acid (65%) and 1 mL hydrofluoric acid (40%) at 190 °C. Second digestion: to neutralize the hydrofluoric acid, was used 10 mL of a 4% boric acid solution at 150 °C	ICP-OES	[81,92]
*Dillenia suffruticosa*, *Dipterocarpus globosus*, *Macaranga trachyphylla*, *Shorea ochracea*	Plant material ashed at 450 °C. The ash is mixed with lithium meta-tetraborate at 1000 °C. The resulting bead was transferred into 10% nitric acid.	ICP-OES	[80,92]
*D. caespitosa*, *Lolium perenne*, *T. aestivum*	100 mg plant material homogenized to a powder; calibration was required.	XRF	[77,92]

ETV, electrothermal vaporization; OID, Oven-Induced Digestion; MBC, molybdenum blue colorimetric; NIRS, Near Infrared Reflectance Spectroscopy; DSi, dissolved Si.

**Table 3:** Extraction techniques from *Medicago sativa* L., *T. aestivum* L., and *P. sativum* L.

Plant	Extraction Technique	Solvent	Conditions	Compounds	Reference
*Medicago sativa* L.	SFE	supercritical carbon dioxide	10 mL of 96% ethanol solution for 3 h at 40, 60, and 80 °C in the dark, and it was incubated in water bath with stirring	phenolics and flavonoids	[10]
*Medicago sativa* L.	Maceration	ethanol	20% aqueous ethanol (*v*/*v*) overnight. After, anhydrous ethanol was added into the soak solution to obtain 75% ethanol solution, pretreated at 50 °C with stirring for 1 h. The extracting solution was filtered, and the filter residue was extracted twice more by following the same steps as above.	flavonoids	[108]
*T. aestivum* L.	HRE	glycerin:water	0.5 g of grounded spelt was subjected to extraction with 5 mL solvent under heat reflux extraction at 60 °C in a water bath for 4 h	phenolics	[7]
	MAE		0.5 g of grounded spelt was subjected to extraction with 5 mL solvent under microwave-assisted extraction for 1 min in a microwave oven (LG MS-197H) at output power 700 W for 30 s		
	UAE		0.5 g of grounded spelt was subjected to extraction with 5 mL solvent under ultrasound-assisted extraction for 30 min in an ultrasonic bath with frequency 50/60 Hz and power 310 W		
*P. sativum* L.	Maceration	acetone-water	700 mL/L aqueous acetone for 30 min attraction of (1:7 *w*/*v*) in a shaking incubator.	phenolics	[109]
*P. sativum* L. (pea flour)	SFE	supercritical carbon dioxide + ethanol extraction	22% ethanol, 86 °C, and 42.71 MPa, 40-min total extraction, including a 10-min static and a 30-min dynamic extraction at a flow rate of 2 mL/min	volatile compounds (1-hexanol, 1-octanol, 1-nonanol, nonanal, and 2-alkyl methoxypyrazines)	[110]

SFE, supercritical fluid extraction; HRE, heat reflux extraction; MAE, microwave-assisted extraction; UAE, ultrasound-assisted extraction.

## Data Availability

Not applicable.

## Abbreviations

AFM	Atomic Force Microscopy
ASE	Accelerated Solvent Extraction
AID	Autoclave Induced Digestion
BEC	Background Equivalent Concentration
DART	Direct Analysis in Real-Time
ETV	Electrothermal Vaporization
FTIR	Fourier Transform Infrared Spectroscopy
GAE	Gallic Acid Equivalents
HPTLC	High-Performance Thin-Layer Chromatography
HRE	Heat Reflux Extraction
HSAB	Hard and Soft Acids and Base Theory
ICP	Inductively Coupled Plasma
IC50	half maximal inhibitory concentration
LA	Laser Ablation
LIBS	Laser-Induced Breakdown Spectrometry
MIC	Minimum Inhibitory Concentration
MALDI MS	Matrix-Assisted Laser Desorption/Ionization Mass Spectrometry
MBC	Molybdenum Blue Colorimetry
MAE	Microwave Assisted Extraction
NMR	Nuclear magnetic resonance
NIRS	Near Infrared Reflectance Spectroscopy
OPLC	Overpressure-Layer Chromatography
OES	Optical Emission Spectrometry
OID	Oven Induced Digestion
PEC	Planar Electro-Chromatography
RF	Radio Frequency
SFE	Supercritical Fluid Extraction
SEM-EDX	Scanning Electron Microscopy with Energy Dispersive X-ray Detector
SEC	Size Exclusion Chromatography
TLC-DB	Thin-Layer Chromatography-Direct Bioautography
TEM	Transmission Electron Microscopy
UAE	Ultrasound-Assisted Extraction
XRF	X-ray Fluorescence

## References

[1] Basu S., Kumar G. (2021). Exploring the significant contribution of silicon in regulation of cellular redox homeostasis for conferring stress tolerance in plants. Plant Physiol. Biochem..

[2] Matichenkov V.V., Bocharnikova E.A. (2001). Chapter 13 The relationship between silicon and soil physical and chemical properties. Silicon in Agriculture.

[3] Ahammed G.J., Yang Y. (2021). Mechanisms of silicon-induced fungal disease resistance in plants. Plant Physiol. Biochem..

[4] Greger M., Landberg T., Vaculík M. (2018). Silicon Influences Soil Availability and Accumulation of Mineral Nutrients in Various Plant Species. Plants.

[5] Luyckx M., Hausman J.-F., Lutts S., Guerriero G. (2017). Silicon and Plants: Current Knowledge and Technological Perspectives. Front. Plant Sci..

[6] Kabata-Pendias A. (2010). Trace Elements in Soils and Plants.

[7] Georgieva-Krasteva L., Hristova I., Mihaylova D., Dobreva K. (2017). Spelt (*Triticum aestivum* ssp. Spelta)—from Field to Cosmetics. Int. J. Pharmacogn. Phytochem. Res..

[8] Rasul M.G. (2018). Extraction, Isolation and Characterization of Natural Products from Medicinal Plants. Int. J. Basic Sci. Appl. Comput..

[9] Guntzer F., Keller C., Meunier J.-D. (2012). Benefits of plant silicon for crops: A review. Agron. Sustain. Dev..

[10] Wrona O., Rafińska K., Walczak-Skierska J., Możeński C., Buszewski B. (2019). Extraction and Determination of Polar Bioactive Compounds from Alfalfa (*Medicago sativa* L.) Using Supercritical Techniques. Molecules.

[11] Waksmundzka-Hajnos M., Sherma J., Kowalska T. (2008). Thin Layer Chromatography in Phytochemistry.

[12] Sasidharan S., Chen Y., Saravanan D., Sundram K.M., Latha L.Y. (2011). Extraction, Isolation And Characterization of Bioactive Compounds from Plants’ Extracts. Afr. J. Trad. Compl. Alt. Med..

[13] Gong J.H., Randall P.D., Flowers J.T. (2006). Silicon deposition in the root reduces sodium uptake in rice (*Oryza sativa* L.) seedlings by reducing bypass flow. Plant Cell Environ..

[14] Ma J.F., Yamaji N. (2006). Silicon uptake and accumulation in higher plants. Trends Plant Sci..

[15] Savant N.K., Korndörfer G.H., Datnoff L.E., Snyder G.H. (1999). Silicon nutrition and sugarcane production: A review. J. Plant Nutr..

[16] Sauer D., Saccone L., Conley D.J., Herrmann L., Sommer M. (2006). Review of methodologies for extracting plant-available and amorphous Si from soils and aquatic sediments. Biogeochemistry.

[17] Drees R.L., Wilding P.L., Smeck E.N., Senkayi L.A., Dixon J., Weed S. (1989). Silica in Soils: Quartz and Disordered Silica Polymorphs. Minerals in Soil Environments.

[18] Schaller J., Puppe D., Kaczorek D., Ellerbrock R., Sommer M. (2021). Silicon Cycling in Soils Revisited. Plants.

[19] Santi L.P., Haris N., Mulyanto D. (2018). Effect of bio-silica on drought tolerance in plants. IOP Conf. Ser. Earth Environ. Sci..

[20] Matichenkov V., Bocharnikova E.A., Calvert D.V., Snyder G.H. (2000). Comparison study of soil silicon status in sandy soils of south Florida. Soil Crop Sci. Soc. Fla. Proc..

[21] Ehrlich H., Demadis K.D., Pokrovsky O.S., Koutsoukos P.G. (2010). Modern Views on Desilicification: Biosilica and Abiotic Silica Dissolution in Natural and Artificial Environments. Chem. Rev..

[22] Bienert G.P., Thorsen M., Schüssler M.D., Nilsson H.R., Wagner A., Tamás M.J., Jahn T.P. (2008). A subgroup of plant aquaporins facilitate the bi-directional diffusion of As(OH)_3_ and Sb(OH)_3_ across membranes. BMC Biol..

[23] Marschner H., Marschner H. (1995). Function of Mineral Nutrients: Micronutrients. Mineral Nutrition of Higher Plants.

[24] Zhu Z., Wei G., Li J., Qian Q., Yu J. (2004). Silicon alleviates salt stress and increases antioxidant enzymes activity in leaves of salt-stressed cucumber (*Cucumis sativus* L.). Plant Sci..

[25] Epstein E. (1999). Silicon. Annu. Rev. Plant Physiol. Plant Mol. Biol..

[26] Perry C.C., Lu Y. (1992). Preparation of silicas from silicon complexes: Role of cellulose in polymerisation and aggregation control. Faraday Trans..

[27] Duboc O., Robbe A., Santner J., Folegnani G., Gallais P., Lecanuet C., Zehetner F., Nagl P., Wenzel W.W. (2019). Silicon Availability from Chemically Diverse Fertilizers and Secondary Raw Materials. Environ. Sci. Technol..

[28] Li Z., Delvaux B. (2019). Phytolith-rich biochar: A potential Si fertilizer in desilicated soils. GCB Bioenergy.

[29] Huang C., Wang L., Gong X., Huang Z., Zhou M., Li J., Wu J., Chang S.X., Jiang P. (2020). Silicon fertilizer and biochar effects on plant and soil PhytOC concentration and soil PhytOC stability and fractionation in subtropical bamboo plantations. Sci. Total Environ..

[30] Xiang T., Ying Y., Teng J., Huang Z., Wu J., Meng C., Jiang P., Tang C., Li J., Zheng R. (2016). Sympodial bamboo species differ in carbon bio-sequestration and stocks within phytoliths of leaf litters and living leaves. Environ. Sci. Pollut. Res..

[31] Liang Y., Liao M., Fang Z., Guo J., Xie X., Xu C. (2021). How silicon fertilizer improves nitrogen and phosphorus nutrient availability in paddy soil?. J. Zhejiang Univ. Sci. B.

[32] Takahashi E., Ma J.F., Miyake Y. (1990). The possibility of silicon as an essential element for higher plants. J. Agric. Food Chem..

[33] Savant N.K., Snyder G.H.S., Datnoff L.E. (1996). Silicon Management and Sustainable Rice Production. Adv. Agron..

[34] Marron A.O., Ratcliffe S., Wheeler G.L., Goldstein R.E., King N., Not F., de Vargas C., Richter D.J. (2016). The Evolution of Silicon Transport in Eukaryotes. Mol. Biol. Evol..

[35] Ma J.F. (2010). Silicon transporters in higher plants. Adv. Exp. Med. Biol..

[36] Ma J.F., Tamai K., Ichii M., Wu G.F. (2002). A Rice Mutant Defective in Si Uptake. Plant Physiol..

[37] Mitani N., Ma J.F., Iwashita T. (2005). Identification of the Silicon Form in Xylem Sap of Rice (*Oryza sativa* L.). Plant Cell Physiol..

[38] Marafon A.C., Endres L. (2013). Silicon: Fertilization and nutrition in higher plants. RCA.

[39] Ranjan A., Sinha R., Bala M., Pareek A., Singla-Pareek S.L., Singh A.K. (2021). Silicon-mediated abiotic and biotic stress mitigation in plants: Underlying mechanisms and potential for stress resilient agriculture. Plant Physiol. Biochem..

[40] Blackman E. (1969). Observations on the development of the silica cells of the leaf sheath of wheat (*Triticum aestivum*). Can. J. Bot..

[41] Dietrich D., Hinke S., Baumann W., Fehlhaber R., Bäucker E., Rühle G., Wienhaus O., Marx G. (2003). Silica accumulation in *Triticum aestivum* L. and *Dactylis glomerata* L. Anal. Bioanal. Chem..

[42] Meunier J.D., Barboni D., Anwar-ul-Haq M., Levard C., Chaurand P., Vidal V., Grauby O., Huc R., Laffont-Schwob I., Rabier J. (2017). Effect of phytoliths for mitigating water stress in durum wheat. New Phytol..

[43] Bennett M.D. (1982). Silicon Deposition in the Roots of *Hordeum sativum* Jess, *Avena sativa* L. and *Triticum aestivum* L. Ann. Bot..

[44] Hodson M.J., Sangster A.G. (1989). Subcellular localization of mineral deposits in the roots of wheat (*Triticum aestivum* L.). Protoplasma.

[45] Casey H.W., Kinrade D.S., Knight G.C.T., Rains W.D., Epstein E. (2004). Aqueous silicate complexes in wheat, *Triticum aestivum* L. Plant Cell Environ..

[46] Howladar S.M., Al-Robai S.A., Al-Zahrani F.S., Howladar M.M., Aldhebiani A.Y. (2018). Silicon and its application method effects on modulation of cadmium stress responses in *Triticum aestivum* (L.) through improving the antioxidative defense system and polyamine gene expression. Ecotoxicol. Environ. Saf..

[47] Shi Z., Yang S., Han D., Zhou Z., Li X., Liu Y., Zhang B. (2018). Silicon alleviates cadmium toxicity in wheat seedlings (*Triticum aestivum* L.) by reducing cadmium ion uptake and enhancing antioxidative capacity. Environ. Sci. Pollut. Res..

[48] Ur Rahman S., Xuebin Q., Zhao Z., Du Z., Imtiaz M., Mehmood F., Hongfei L., Hussain B., Ashraf M.N. (2021). Alleviatory effects of Silicon on the morphology, physiology, and antioxidative mechanisms of wheat (*Triticum aestivum* L.) roots under cadmium stress in acidic nutrient solutions. Sci. Rep..

[49] Rizwan M., Meunier J.-D., Miche H., Keller C. (2012). Effect of silicon on reducing cadmium toxicity in durum wheat (*Triticum turgidum* L. cv. Claudio W.) grown in a soil with aged contamination. J. Hazard. Mater..

[50] Greger M., Kabir A.H., Landberg T., Maity P.J., Lindberg S. (2016). Silicate reduces cadmium uptake into cells of wheat. Environ. Pollut..

[51] Thind S., Hussain I., Ali S., Rasheed R., Ashraf M.A. (2021). Silicon Application Modulates Growth, Physio-Chemicals, and Antioxidants in Wheat (*Triticum aestivum* L.) Exposed to Different Cadmium Regimes. Dose-Response.

[52] Wu J., Geilfus C.-M., Pitann B., Mühling K.-H. (2016). Silicon-enhanced oxalate exudation contributes to alleviation of cadmium toxicity in wheat. Environ. Exp. Bot..

[53] Ur Rahman S., Xuebin Q., Yasin G., Cheng H., Mehmood F., Zain M., Shehzad M., Ahmad M.I., Riaz L., Rahim A. (2021). Role of silicon on root morphological characters of wheat (*Triticum aestivum* L.) plants grown under Cd-contaminated nutrient solution. Acta Physiol. Plant.

[54] Gong H., Zhu X., Chen K., Wang S., Zhang C. (2005). Silicon alleviates oxidative damage of wheat plants in pots under drought. Plant Sci..

[55] Pei Z.F., Ming D.F., Liu D., Wan G.L., Geng X.X., Gong H.J., Zhou W.J. (2010). Silicon Improves the Tolerance to Water-Deficit Stress Induced by Polyethylene Glycol in Wheat (*Triticum aestivum* L.) Seedlings. J. Plant Growth Regul..

[56] Alzahrani Y., Kuşvuran A., Alharby H.F., Kuşvuran S., Rady M.M. (2018). The defensive role of silicon in wheat against stress conditions induced by drought, salinity or cadmium. Ecotoxicol. Environ. Saf..

[57] Silva I.T., Rodrigues F.Á., Oliveira J.R., Pereira S.C., Andrade C.C.L., Silveira P.R., Conceição M.M. (2010). Wheat Resistance to Bacterial Leaf Streak Mediated by Silicon. J. Phytopathol..

[58] Goussain M.M., Prado E., Moraes J.C. (2005). Effect of silicon applied to wheat plants on the biology and probing behaviour of the greenbug *Schizaphis graminum* (Rond.) (Hemiptera: Aphididae). Neotrop. Entomol..

[59] Parry W.D., Winslow A. (1977). Electron-probe Microanalysis of Silicon Accumulation in the Leaves and Tendrils of *Pisum sativum* (L.) Following Root Severance. Ann. Bot..

[60] Merwad A.-R.M.A. (2018). Response of yield and nutrients uptake of pea plants to silicate under sandy soil conditions. Commun. Soil Sci. Plant Anal..

[61] Tripathi D.K., Singh V.P., Prasad S.M., Chauhan D.K., Dubey N.K. (2015). Silicon nanoparticles (SiNp) alleviate chromium (VI) phytotoxicity in *Pisum sativum* (L.) seedlings. Plant Physiol. Biochem..

[62] Rahman M.F., Ghosal A., Alam M.F., Kabir A.H. (2017). Remediation of cadmium toxicity in field peas (*Pisum sativum* L.) through exogenous silicon. Ecotoxicol. Environ. Saf..

[63] Jan S., Alyemeni M.N., Wijaya L., Alam P., Siddique K.H., Ahmad P. (2018). Interactive effect of 24-epibrassinolide and silicon alleviates cadmium stress via the modulation of antioxidant defense and glyoxalase systems and macronutrient content in *Pisum sativum* L. seedlings. BMC Plant Biol..

[64] Feng Y., Li X., Guo S., Chen X., Chen T., He Y., Shabala S., Yu M. (2019). Extracellular silica nanocoat formed by layer-by-layer (LBL) self-assembly confers aluminum resistance in root border cells of pea (*Pisum sativum*). J. Nanobiotechnol..

[65] Arafa S.A., Attia K.A., Niedbała G., Piekutowska M., Alamery S., Abdelaal K., Alateeq T.K., Ali M.A.M., Elkelish A., Attallah S.Y. (2021). Seed Priming Boost Adaptation in Pea Plants under Drought Stress. Plants.

[66] Dann E.K., Muir S. (2002). Peas grown in media with elevated plant-available silicon levels have higher activities of chitinase and β-1,3-glucanase, are less susceptible to a fungal leaf spot pathogen and accumulate more foliar silicon. Australas. Plant Pathol..

[67] Głowacka K., Szultka-Młyńska M., Cichorek M., Orzoł A., Rogowska A., Cruzado Tafur E., Pomastowski P., Górecki R., Buszewski B. (2022). Znaczenie krzemu dla wybranych gatunków roślin. Kosmos.

[68] Sahebi M., Hanafi M.M., Akmar A.S.N., Rafii M.Y., Azizi P., Tengoua F.F., Azwa J.N.M., Shabanimofrad M. (2015). Importance of silicon and mechanisms of biosilica formation in plants. BioMed Res. Int..

[69] Gallego S.M., Pena L.B., Barcia R.A., Azpilicueta C.E., Iannone M.F., Rosales E.P., Zawoznik M.S., Groppa M.D., Benavides M.P. (2012). Unravelling cadmium toxicity and tolerance in plants: Insight into regulatory mechanisms. Environ. Exp. Bot..

[70] Haider F.U., Liqun C., Coulter J.A., Cheema S.A., Wu J., Zhang R., Wenjun M., Farooq M. (2021). Cadmium toxicity in plants: Impacts and remediation strategies. Ecotoxicol. Environ. Saf..

[71] Liu H., Guo Z.G. (2013). Forage Yield and Water Use Efficiency of Alfalfa Applied with Silicon under Water Deficit Conditions. Philipp. Agric..

[72] Kabir A.H., Hossain M.M., Khatun M.A., Mandal A., Haider S.A. (2016). Role of Silicon Counteracting Cadmium Toxicity in Alfalfa (*Medicago sativa* L.). Front. Plant Sci..

[73] Liu D., Liu M., Liu X.-L., Cheng X.-G., Liang Z.-W. (2018). Silicon Priming Created an Enhanced Tolerance in Alfalfa (*Medicago sativa* L.) Seedlings in Response to High Alkaline Stress. Front. Plant Sci..

[74] Meng Y., Yin Q., Yan Z., Wang Y., Niu J., Zhang J., Fan K. (2020). Exogenous Silicon Enhanced Salt Resistance by Maintaining K^+^/Na^+^ Homeostasis and Antioxidant Performance in Alfalfa Leaves. Front. Plant Sci..

[75] El Moukhtari A., Carol P., Mouradi M., Savoure A., Farissi M. (2021). Silicon improves physiological, biochemical, and morphological adaptations of alfalfa (*Medicago sativa* L.) during salinity stress. Symbiosis.

[76] Namieśnik J., Szefer P. (2008). Preparing samples for analysis—The key to analytical success. Ecol. Chem. Eng. S-Chem. I Inz. Ekol. S.

[77] Reidinger S., Ramsey M.H., Hartley S.E. (2012). Rapid and accurate analyses of silicon and phosphorus in plants using a portable X-ray fluorescence spectrometer. New Phytol..

[78] Masson P., Dalix T., Bussière S. (2010). Determination of Major and Trace Elements in Plant Samples by Inductively Coupled Plasma–Mass Spectrometry. Commun. Soil Sci. Plant Anal..

[79] Li Z., Song Z., Singh B.P., Wang H. (2019). The impact of crop residue biochars on silicon and nutrient cycles in croplands. Sci. Total Environ..

[80] Nakamura R., Cornelis J.-T., de Tombeur F., Nakagawa M., Kitajima K. (2020). Comparative analysis of borate fusion versus sodium carbonate extraction for quantification of silicon contents in plants. J. Plant Res..

[81] Puppe D., Höhn A., Kaczorek D., Wanner M., Wehrhan M., Sommer M. (2017). How big is the influence of biogenic silicon pools on short-term changes in water-soluble silicon in soils? Implications from a study of a 10-year-old soil–plant system. Biogeosciences.

[82] Barros J.A.V.A., de Souza P.F., Schiavo D., Nóbrega J.A. (2016). Microwave-assisted digestion using diluted acid and base solutions for plant analysis by ICP OES. J. Anal. At. Spectrom..

[83] Guntzer F., Keller C., Meunier J.D. (2010). Determination of the silicon concentration in plant material using Tiron extraction. New Phytol..

[84] Clymans W., Conley D.J., Battles J.J., Frings P.J., Koppers M.M., Likens G.E., Johnson C.E. (2016). Silica uptake and release in live and decaying biomass in a northern hardwood forest. Ecology.

[85] Snyder G.H. (2001). Chapter 11 Methods for silicon analysis in plants, soils, and fertilizers. Silicon in Agriculture.

[86] Smis A., Ancin Murguzur F.J., Struyf E., Soininen E.M., Herranz Jusdado J.G., Meire P., Bråthen K.A. (2014). Determination of plant silicon content with near infrared reflectance spectroscopy. Front. Plant Sci..

[87] Taber H.G., Shogren D., Lu G. (2002). Extraction of silicon from plant tissue with dilute HCl and HF and measurement by modified inductive coupled argon plasma procedures. Commun. Soil Sci. Plant Anal..

[88] Masson P., Dauthieu M., Trolard F., Denaix L. (2007). Application of direct solid analysis of plant samples by electrothermal vaporization-inductively coupled plasma atomic emission spectrometry: Determination of Cd and Si for environmental purposes. Spectrochim. Acta Part B At. Spectrosc..

[89] Babu T., Tubana B., Paye W., Kanke Y., Datnoff L. (2016). Establishing Soil Silicon Test Procedure and Critical Silicon Level for Rice in Louisiana Soils. Commun. Soil Sci. Plant Anal..

[90] Kraska J.E., Breitenbeck G.A. (2010). Survey of the Silicon Status of Flooded Rice in Louisiana. Agron. J..

[91] Paye W.S. (2019). Silicon Fertilization in Rice and Wheat: Dynamics with Trace Elements and Effect of Silicate Slag Granular Size on the Release Pattern of Monosilicic Acid in Soil. Ph.D. Thesis.

[92] Katz O., Puppe D., Kaczorek D., Prakash N.B., Schaller J. (2021). Silicon in the Soil-Plant Continuum: Intricate Feedback Mechanisms within Ecosystems. Plants.

[93] Mitra A., Rimstidt J.D. (2009). Solubility and dissolution rate of silica in acid fluoride solutions. Geochim. Cosmochim. Acta.

[94] Saito K., Yamamoto A., Sa T., Saigusa M. (2005). Rapid, Micro-Methods to Estimate Plant Silicon Content by Dilute Hydrofluoric Acid Extraction and Spectrometric Molybdenum Method. Soil Sci. Plant Nutr..

[95] Santos M.A., Silva A.B.S., Machado R.C., Dias E.A., Barros J.A., Nogueira A.R.A. (2020). Silicon determination by microwave-induced plasma optical emission spectrometry: Considerations and strategies for the use of tetrafluorboric acid and sodium hydroxide in sample preparation procedures. Spectrochim. Acta Part B At. Spectrosc..

[96] He C., Ma J., Wang L. (2015). A hemicellulose-bound form of silicon with potential to improve the mechanical properties and regeneration of the cell wall of rice. New Phytol..

[97] Gholami M., Behkami S., Zain S.M., Bakirdere S. (2016). A simple design for microwave assisted digestion vessel with low reagent consumption suitable for food and environmental samples. Sci. Rep..

[98] Xiao Z., Peng M., Mei Y., Tan L., Liang Y. (2021). Effect of organosilicone and mineral silicon fertilizers on chemical forms of cadmium and lead in soil and their accumulation in rice. Environ. Pollut..

[99] Bokor B., Soukup M., Vaculík M., Vd’ačný P., Weidinger M., Lichtscheidl I., Vávrová S., Šoltys K., Sonah H., Deshmukh R. (2019). Silicon Uptake and Localisation in Date Palm (*Phoenix dactylifera*)—A Unique Association with Sclerenchyma. Front. Plant Sci..

[100] Kido N., Yokoyama R., Yamamoto T., Furukawa J., Iwai H., Satoh S., Nishitani K. (2015). The matrix polysaccharide (1;3,1;4)-β-D-glucan is involved in silicon-dependent strengthening of rice cell wall. Plant Cell Physiol..

[101] Kopittke P.M., Gianoncelli A., Kourousias G., Green K., McKenna B.A. (2017). Alleviation of Al Toxicity by Si Is Associated with the Formation of Al–Si Complexes in Root Tissues of Sorghum. Front. Plant Sci..

[102] Bowen J.E. (1972). Manganese-silicon interaction and its effect on growth of Sudan grass. Plant Soil.

[103] Pan T., Zhang J., He L., Hafeez A., Ning C., Cai K. (2021). Silicon Enhances Plant Resistance of Rice against Submergence Stress. Plants.

[104] Raid R.N., Anderson D.L., Ulloa M.F. (1992). Influence of cultivar and amendment of soil with calcium silicate slag on foliar disease development and yield of sugar-cane. Crop Prot..

[105] Azwanida N.N. (2015). A Review on the Extraction Methods Use in Medicinal Plants, Principle, Strength and Limitation. Med. Aromat. Plants.

[106] Ojha K.S., Aznar R., O’Donnell C., Tiwari B.K. (2020). Ultrasound technology for the extraction of biologically active molecules from plant, animal and marine sources. Trends Anal. Chem..

[107] Jiang T., Ghosh R., Charcosset C. (2021). Extraction, purification and applications of curcumin from plant materials-A comprehensive review. Trends Food Sci. Technol..

[108] Chen S., Li X., Liu X., Wang N., An Q., Ye X.M., Zhao Z.T., Zhao M., Han Y., Ouyang K.H. (2020). Investigation of Chemical Composition, Antioxidant Activity, and the Effects of Alfalfa Flavonoids on Growth Performance. Oxid. Med. Cell. Longev..

[109] Troszyńska A., Estrella I., López-Amóres M., Hernández T. (2002). Antioxidant Activity of Pea (*Pisum sativum* L.) Seed Coat Acetone Extract. LWT-Food Sci. Technol..

[110] Vatansever S., Xu M., Magallanes-López A., Chen B., Hall C. (2021). Supercritical Carbon Dioxide + Ethanol Extraction to Improve Organoleptic Attributes of Pea Flour with Applications of Sensory Evaluation, HS-SPME-GC, and GC-Olfactory. Processes.

[111] Tambun R., Alexander V., Ginting Y. (2021). Performance comparison of maceration method, soxhletation method, and microwave-assisted extraction in extracting active compounds from soursop leaves (Annona muricata): A review. IOP Conf. Ser. Mater. Sci. Eng..

[112] Sadowska B., Budzyńska A., Więckowska-Szakiel M., Paszkiewicz M., Stochmal A., Moniuszko-Szajwaj B., Kowalczyk M., Różalska B. (2014). New pharmacological properties of *Medicago sativa* and *Saponaria officinalis* saponin-rich fractions addressed to *Candida albicans*. J. Med. Microbiol..

[113] Rafińska K., Pomastowski P., Wrona O., Górecki R., Buszewski B. (2017). *Medicago sativa* as a source of secondary metabolites for agriculture and pharmaceutical industry. Phytochem. Lett..

[114] Zhang Q.-W., Lin L.-G., Ye W.-C. (2018). Techniques for extraction and isolation of natural products: A comprehensive review. Chin. Med..

[115] Tava A., Mella M., Avato P., Biazzi E., Pecetti L., Bialy Z., Jurzysta M. (2009). New Triterpenic Saponins from the Aerial Parts of *Medicago arabica* (L.) Huds. J. Agric. Food Chem..

[116] Kiełbasa A., Krakowska-Sieprawska A., Kowalkowski T., Rafińska K., Buszewski B. (2020). Distribution of sapogenins in morphological *Medicago sativa* L. parts: Comparison of various extraction techniques. J. Sep. Sci..

[117] Khaledi M., Khaledi F., Asadi-Samani M., Gholipour A., Mahmoodi Kouhi A. (2018). Phytochemical evaluation and antibacterial effects of *Medicago sativa*, *Onosma sericeum*, *Parietaria judaica* L., *Phlomis persica* and *Echinophora platyloba* DC. on *Enterococcus faecalis*. Biomed. Res. Ther..

[118] Amber R., Adnan M., Tariq A., Khan S.N., Mussarat S., Hashem A., Al-huqail A.A., Al-Arjani A.-B.F., Abd_Allah E.F. (2018). Antibacterial activity of selected medicinal plants of northwest Pakistan traditionally used against mastitis in livestock. Saudi J. Biol. Sci..

[119] Chegini H., Oshaghi M., Boshagh M.A., Foroutan P., Jahangiri A.H. (2018). Antibacterial effect of *Medicago sativa* extract on the common bacterial in sinusitis infection. IJBMPH.

[120] Golla K., Vutukuru S.S., Rani J.U., Meghanath P., Pasha C. (2016). Screening of small peptides from various germinating seeds having antimicrobial activity. IOSR J. Pharm. Biol. Sci..

[121] Pagnussatt F.A., Bretanha C.C., Kupski L., Garda-Buffon J., Badiale-Furlong E. (2013). Promising Antifungal Effect of Rice (*Oryza sativa* L.), Oat (*Avena sativa* L.) and Wheat (*Triticum aestivum* L.) Extracts. J. Appl. Biotechnol. Rep..

[122] Rodrigues F., Palmeira-de-Oliveira A., das Neves J., Sarmento B., Amaral M.H., Oliveira M.B. (2013). *Medicago* spp. extracts as promising ingredients for skin care products. Ind. Crops Prod..

[123] Sangwan Y.S., Hooda T., Kumar H. (2013). Antibacterial, Antioxidant, Antimicrobial And Wound Healing Potential of *Triticum aestivum* & *Terminalia bellirica*. Int. J. Pharma Prof. Res..

[124] Hadrich F., Arbi M.E., Boukhris M., Sayadi S., Cherif S. (2014). Valorization of the Peel of Pea: *Pisum sativum* by Evaluation of Its Antioxidant and Antimicrobial Activities. J. Oleo Sci..

[125] Sundaresan A., Selvi A., Manonmani H.K. (2015). The Anti-Microbial Properties of *Triticum aestivum* (Wheat Grass) Extract. Int. J. Biotech. Well. Indus..

[126] Kim M.-H., Jo S.-H., Ha K.-S., Song J.-H., Jang H.-D., Kwon Y.-I. (2010). Antimicrobial activities of 1,4-benzoquinones and wheat germ extract. J. Microbiol. Biotechnol..

[127] Nair S.S., Madembil N.C., Nair P., Raman S., Veerabadrappa S.B. (2013). Comparative analysis of the antibacterial activity of some phytolectins. Int. Curr. Pharm. J..

[128] Narendhirakannan R.T., Nirmala J.G., Caroline A., Lincy S., Saj M., Durai D. (2012). Evaluation of antibacterial, antioxidant and wound healing properties of seven traditional medicinal plants from India in experimental animals. Asian Pac. J. Trop. Biomed..

[129] Caunii A., Pribac G., Grozea I., Gaitin D., Samfira I. (2012). Design of optimal solvent for extraction of bio-active ingredients from six varieties of *Medicago sativa*. Chem. Cent. J..

[130] Bialy Z., Jurzysta M., Mella M., Tava A. (2004). Triterpene saponins from aerial parts of *Medicago arabica* L. J. Agric. Food Chem..

[131] Avato P., Bucci R., Tava A., Vitali C., Rosato A., Bialy Z., Jurzysta M. (2006). Antimicrobial activity of saponins from *Medicago* sp.: Structure-activity relationship. Phytother. Res..

[132] Abbruscato P., Tosi S., Crispino L., Biazzi E., Menin B., Picco A.M., Pecetti L., Avato P., Tava A. (2014). Triterpenoid Glycosides from *Medicago sativa* as Antifungal Agents against *Pyricularia oryzae*. J. Agric. Food Chem..

[133] Chaves J.O., de Souza M.C., Da Silva L.C., Lachos-Perez D., Torres-Mayanga P.C., Da Machado A.P.F., Forster-Carneiro T., Vázquez-Espinosa M., González-de-Peredo A.V., Barbero G.F. (2020). Extraction of Flavonoids From Natural Sources Using Modern Techniques. Front. Chem..

[134] Povilaitis D., Šulniūtė V., Venskutonis P.R., Kraujalienė V. (2015). Antioxidant properties of wheat and rye bran extracts obtained by pressurized liquid extraction with different solvents. J. Cereal Sci..

[135] Abdel-Aal E.-S.M., Akhtar H., Rabalski I., Bryan M. (2014). Accelerated, Microwave-Assisted, and Conventional Solvent Extraction Methods Affect Anthocyanin Composition from Colored Grains. J. Food Sci..

[136] Liyana-Pathirana C., Shahidi F. (2005). Optimization of extraction of phenolic compounds from wheat using response surface methodology. Food Chem..

[137] Chen H., Ji A., Qiu S., Liu Y., Zhu Q., Yin L. (2018). Covalent conjugation of bovine serum album and sugar beet pectin through Maillard reaction/laccase catalysis to improve the emulsifying properties. Food Hydrocoll..

[138] Krakowska A., Rafińska K., Walczak J., Kowalkowski T., Buszewski B. (2017). Comparison of Various Extraction Techniques of *Medicago sativa*: Yield, Antioxidant Activity, and Content of Phytochemical Constituents. J. AOAC Int..

[139] Jäpelt R.B., Jakobsen J. (2016). Analysis of vitamin K1 in fruits and vegetables using accelerated solvent extraction and liquid chromatography tandem mass spectrometry with atmospheric pressure chemical ionization. Food Chem..

[140] Hedrick J.L., Mulcahey L.J., Taylor L.T. (1992). Supercritical fluid extraction. Microchim. Acta.

[141] Athukorala Y., Hosseinian F.S., Mazza G. (2010). Extraction and fractionation of alkylresorcinols from triticale bran by two-step supercritical carbon dioxide. LWT-Food Sci. Technol..

[142] Krakowska-Sieprawska A., Rafińska K., Walczak-Skierska J., Kiełbasa A., Buszewski B. (2021). Promising Green Technology in Obtaining Functional Plant Preparations: Combined Enzyme-Assisted Supercritical Fluid Extraction of Flavonoids Isolation from *Medicago Sativa* Leaves. Materials.

[143] Krakowska A., Rafińska K., Walczak J., Buszewski B. (2018). Enzyme-assisted optimized supercritical fluid extraction to improve *Medicago sativa* polyphenolics isolation. Ind. Crops Prod..

[144] Rodrigues V.H., de Melo M.M., Portugal I., Silva C.M. (2021). Lupane-type triterpenoids from *Acacia dealbata* bark extracted by different methods. Ind. Crops Prod..

[145] Zaniol F., Calisto J.F., Cozzer G., Ferro D.M., Dias J.L., Rodrigues L.G., Mazzutti S., Rezende R.S., Simões D.A., Ferreira S.R. (2020). Comparative larvicidal effect of *Pterodon* spp. extracts obtained by different extraction methods. J. Supercrit. Fluids.

[146] Favareto R., Teixeira M.B., Soares F.A.L., Belisário C.M., Corazza M.L., Cardozo-Filho L. (2017). Study of the supercritical extraction of *Pterodon* fruits (Fabaceae). J. Supercrit. Fluids.

[147] Bogdanovic A., Tadic V., Stamenic M., Petrovic S., Skala D. (2016). Supercritical carbon dioxide extraction of *Trigonella foenum-graecum* L. seeds: Process optimization using response surface methodology. J. Supercrit. Fluids.

[148] Ge Y., Yan H., Hui B., Ni Y., Wang S., Cai T. (2002). Extraction of Natural Vitamin E from Wheat Germ by Supercritical Carbon Dioxide. J. Agric. Food Chem..

[149] Piras A., Rosa A., Falconieri D., Porcedda S., Dessì M., Marongiu B. (2009). Extraction of Oil from Wheat Germ by Supercritical CO_2_. Molecules.

[150] Ballard T.S., Mallikarjunan P., Zhou K., O’Keefe S. (2010). Microwave-assisted extraction of phenolic antioxidant compounds from peanut skins. Food Chem..

[151] Oufnac D.S., Xu Z., Sun T., Sabliov C., Prinyawiwatkul W., Godber J.S. (2007). Extraction of Antioxidants from Wheat Bran Using Conventional Solvent and Microwave-Assisted Methods. Cereal Chem..

[152] Crundwell F.K. (2017). On the Mechanism of the Dissolution of Quartz and Silica in Aqueous Solutions. ACS Omega.

[153] Guerra M., Adame A., de Almeida E., Brasil M., Schaefer C., Krug F. (2018). In situ Determination of K, Ca, S and Si in Fresh Sugar Cane Leaves by Handheld Energy Dispersive X-Ray Fluorescence Spectrometry. J. Braz. Chem. Soc..

[154] Thorne S.J., Hartley S.E., Maathuis F.J.M. (2021). The Effect of Silicon on Osmotic and Drought Stress Tolerance in Wheat Landraces. Plants.

[155] van der Ent A., Casey L.W., Blamey F.P.C., Kopittke P.M. (2020). Time-resolved laboratory micro-X-ray fluorescence reveals silicon distribution in relation to manganese toxicity in soybean and sunflower. Ann. Bot..

[156] Michel B.E. (1983). Evaluation of the water potentials of solutions of polyethylene glycol 8000 both in the absence and presence of other solutes. Plant Physiol..

[157] Johnson J. (2014). Accurate Measurements of Low Z Elements in Sediments and Archaeological Ceramics Using Portable X-ray Fluorescence (PXRF). J. Archaeol. Method Theory.

[158] Deshmukh R.K., Vivancos J., Guérin V., Sonah H., Labbé C., Belzile F., Bélanger R.R. (2013). Identification and functional characterization of silicon transporters in soybean using comparative genomics of major intrinsic proteins in Arabidopsis and rice. Plant Mol. Biol..

[159] Baranowski R., Rybak A., Baranowska I. (2002). Speciation analysis of elements in soil samples by XRF. Pol. J. Environ. Stud..

[160] Frick D.A., Schuessler J.A., Sommer M., Blanckenburg F. (2019). Laser Ablation In Situ Silicon Stable Isotope Analysis of Phytoliths. Geostand. Geoanalytical Res..

[161] Fleck A.T., Schulze S., Hinrichs M., Specht A., Waßmann F., Schreiber L., Schenk M.K. (2015). Silicon Promotes Exodermal Casparian Band Formation in Si-Accumulating and Si-Excluding Species by Forming Phenol Complexes. PLoS ONE.

[162] Chen G., Taherymoosavi S., Cheong S., Yin Y., Akter R., Marjo C.E., Rich A.M., Mitchell D.R.G., Fan X., Chew J. (2021). Advanced characterization of biomineralization at plaque layer and inside rice roots amended with iron- and silica-enhanced biochar. Sci. Rep..

[163] Tripathi D.K., Kumar R., Pathak A.K., Chauhan D.K., Rai A.K. (2012). Laser-Induced Breakdown Spectroscopy and Phytolith Analysis: An Approach to Study the Deposition and Distribution Pattern of Silicon in Different Parts of Wheat (*Triticum aestivum* L.) Plant. Agric. Res..

[164] Soukup M., Martinka M., Bosnić D., Čaplovičová M., Elbaum R., Lux A. (2017). Formation of silica aggregates in sorghum root endodermis is predetermined by cell wall architecture and development. Ann. Bot..

[165] Quigley K.M., Althoff A.G., Donati G.L. (2016). Inductively coupled plasma optical emission spectrometry as a reference method for silicon estimation by near infrared spectroscopy and potential application to global-scale studies of plant chemistry. Microchem. J..

[166] Sucharová J., Suchara I. (2006). Determination of 36 elements in plant reference materials with different Si contents by inductively coupled plasma mass spectrometry: Comparison of microwave digestions assisted by three types of digestion mixtures. Anal. Chim. Acta.

[167] Le Blond J.S., Strekopytov S., Unsworth C., Williamson B.J. (2011). Testing a new method for quantifying Si in silica-rich biomass using HF in a closed vessel microwave digestion system. Anal. Methods.

[168] Ramírez-Olvera S.M., Trejo-Téllez L.I., Gómez-Merino F.C., Del Ruíz-Posadas L.M., Alcántar-González E.G., Saucedo-Veloz C. (2021). Silicon Stimulates Plant Growth and Metabolism in Rice Plants under Conventional and Osmotic Stress Conditions. Plants.

[169] Gu H.-H., Zhan S.-S., Wang S.-Z., Tang Y.-T., Chaney R.L., Fang X.-H., Cai X.-D., Qiu R.-L. (2012). Silicon-mediated amelioration of zinc toxicity in rice (*Oryza sativa* L.) seedlings. Plant Soil.

[170] May T.W., Wiedmeyer R.H. (1998). A Table of Polyatomic Interferences in ICP-MS. At. Spectrosc..

[171] Mihaylova V., Lyubomirova V., Djingova R. (2013). Optimization of sample preparation and ICP-MS analysis for determination of 60 elements for characterization of the plant ionome. Int. J. Environ. Anal. Chem..

[172] Feng X., Wu S., Wharmby A., Wittmeier A. (1999). Microwave digestion of plant and grain standard reference materials in nitric and hydrofluoric acids for multi-elemental determination by inductively coupled plasma mass spectrometry. J. Anal. At. Spectrom..

[173] Aureli F., Ciprotti M., D’Amato M., Da do Nascimento Silva E., Nisi S., Passeri D., Sorbo A., Raggi A., Rossi M., Cubadda F. (2020). Determination of Total Silicon and SiO_2_ Particles Using an ICP-MS Based Analytical Platform for Toxicokinetic Studies of Synthetic Amorphous Silica. Nanomaterials.

[174] Lum T.-S., Leung K.S.-Y. (2016). Strategies to overcome spectral interference in ICP-MS detection. J. Anal. At. Spectrom..

[175] Liu H., Jiang S.-J. (2003). Dynamic reaction cell inductively coupled plasma mass spectrometry for determination of silicon in steel. Spectrochim. Acta Part B At. Spectrosc..

[176] Klemens P., Heumann K.G. (2001). Development of an ICP–HRIDMS method for accurate determination of traces of silicon in biological and clinical samples. Fresenius J. Anal. Chem..

[177] Mitani N., Ma J.F. (2005). Uptake system of silicon in different plant species. J. Exp. Bot..

[178] Liang Y., Hua H., Zhu Y.-G., Zhang J., Cheng C., Römheld V. (2006). Importance of plant species and external silicon concentration to active silicon uptake and transport. New Phytol..

[179] Hodson M.J., White P.J., Mead A., Broadley M.R. (2005). Phylogenetic variation in the silicon composition of plants. Ann. Bot..

[180] Reis L.S., Gonçalves É.C.B.d.A. (2015). Chemical speciation: An instrument for evaluation of mineral bioavailability. Cienc. Rural.

[181] Schmitt C., Aberkane L., Sanchez C., Phillips G.O., Williams P.A. (2009). Protein-polysaccharide complexes and coacervates. Handbook of Hydrocolloids.

[182] Ebdon L., Foulkes M., Fredeen K., Hanna C., Sutton K. (1998). Silicon speciation using reversed phase high performance liquid chromatography-inductively coupled plasma atomic emission spectrometry—Radial versus axial viewing. Spectrochim. Acta Part B At. Spectrosc..

[183] Carter J., Ebdon L., Hywel Evans E. (2004). Speciation of silicon and phosphorous using liquid chromatography coupled with sector field high resolution ICP-MS. Microchem. J..

[184] Ma J.F. (2004). Role of silicon in enhancing the resistance of plants to biotic and abiotic stresses. Soil Sci. Plant Nutr..

[185] Park J.-J., Kim K.W., Park T.-J., Park E.W., Kim Y. (2006). Solid-state NMR spectroscopy of silicon-treated rice with enhanced host resistance against blast. Anal. Sci..

[186] Schaller J., Brackhage C., Paasch S., Brunner E., Bäucker E., Dudel E.G. (2013). Silica uptake from nanoparticles and silica condensation state in different tissues of *Phragmites australis*. Sci. Total Environ..

[187] Cabrera Y., Cabrera A., Larsen F.H., Felby C. (2016). Solid-state 29Si NMR and FTIR analyses of lignin-silica coprecipitates. Holzforschung.

[188] Atia A.A., El-Nahas A.M., Marie A.M., Mahdy L.D.A. (2006). Adsorption of Oleic Acid on Silica Gel Derived from Rice Ash Hulls: Experimental and Theoretical Studies. Adsorpt. Sci. Technol..

[189] Kinrade S.D., Del Nin J.W., Schach A.S., Sloan T.A., Wilson K.L., Knight C.T.G. (1999). Stable Five- and Six-Coordinated Silicate Anions in Aqueous Solution. Science.

[190] Stamm F.M., Méheut M., Zambardi T., Chmeleff J., Schott J., Oelkers E.H. (2020). Extreme silicon isotope fractionation due to Si organic complexation: Implications for silica biomineralization. Earth Planet. Sci. Lett..

[191] Pu J., Wang L., Zhang W., Ma J., Zhang X., Putnis C.V. (2021). Organically-bound silicon enhances resistance to enzymatic degradation and nanomechanical properties of rice plant cell walls. Carbohydr. Polym..

[192] Korndörfer G.H., Snyder G.H., Ulloa M., Powell G., Datnoff L.E. (2001). Calibration of soil and plant silicon analysis for rice production. J. Plant Nutr..

[193] Inanaga S., Okasaka A., Tanaka S. (1995). Does silicon exist in association with organic compounds in rice plant?. Soil Sci. Plant Nutr..

[194] Matychenkov I.V., Khomyakov D.M., Pakhnenko E.P., Bocharnikova E.A., Matychenkov V.V. (2016). Mobile Si-rich compounds in the soil–plant system and methods for their determination. Mosc. Univ. Soil Sci. Bull..

[195] Currie H.A., Perry C.C. (2007). Silica in Plants: Biological, Biochemical and Chemical Studies. Ann. Bot..

[196] Guerriero G., Hausman J.-F., Legay S. (2016). Silicon and the Plant Extracellular Matrix. Front. Plant Sci..

[197] Ishii T., Matsunaga T. (2008). Aqueous Macromolecules with Silicon from Alcoholinsoluble Residues of Rice Seedlings. Jpn. Agric. Res. Q..

[198] Sahebi M., Hanafi M.M., Abdullah S.N.A., Rafii M.Y., Azizi P., Nejat N., Idris A.S. (2014). Isolation and Expression Analysis of Novel Silicon Absorption Gene from Roots of Mangrove (*Rhizophora apiculata*) via Suppression Subtractive Hybridization. BioMed Res. Int..

[199] Kauss H., Seehaus K., Franke R., Gilbert S., Dietrich R.A., Kröger N. (2003). Silica deposition by a strongly cationic proline-rich protein from systemically resistant cucumber plants. Plant J..

[200] Shimizu K., Del Amo Y., Brzezinski M.A., Stucky G.D., Morse D.E. (2001). A novel fluorescent silica tracer for biological silicification studies. Chem. Biol..

[201] Dabney C., Ostergaard J., Watkins E., Chen C. (2016). A novel method to characterize silica bodies in grasses. Plant Methods.

[202] Blecher I.C., Seidel R., Thomann R., Speck T. (2012). Comparison of Different Methods for the Detection of Silica Inclusions in Plant Tissues. Int. J. Plant Sci..

[203] De Cassia Félix Alvarez R., de Mello Prado R., Felisberto G., Deus A.C.F., Oliveira R. (2018). Effects of Soluble Silicate and Nanosilica Application on Rice Nutrition in an Oxisol. Pedosphere.

[204] Głazowska S., Baldwin L., Mravec J., Bukh C., Hansen T.H., Jensen M.M., Fangel J.U., Willats W.G.T., Glasius M., Felby C. (2018). The impact of silicon on cell wall composition and enzymatic saccharification of *Brachypodium distachyon*. Biotechnol. Biofuels..

[205] Hodson M.J., Sangster A.G. (1993). The Interaction Between Silicon and Aluminium in *Sorghum bicolor* (L.) Moench: Growth Analysis and X-ray Microanalysis. Ann. Bot..

[206] Zexer N., Elbaum R. (2020). Unique lignin modifications pattern the nucleation of silica in sorghum endodermis. J. Exp. Bot..

[207] Asgari F., Majd A., Jonoubi P., Najafi F. (2018). Effects of silicon nanoparticles on molecular, chemical, structural and ultrastructural characteristics of oat (*Avena sativa* L.). Plant Physiol. Biochem..

[208] Perry C.C., Williams R.J., Fry S.C. (1987). Cell Wall Biosynthesis during Silicification of Grass Hairs. J. Plant Physiol..

[209] Perry C.C., Fraser M.A. (1991). Silica Deposition and Ultrastructure in the Cell Wall of *Equisetum arvense*: The Importance of Cell Wall Structures and Flow Control in Biosilicification?. Philos. Trans. R. Soc. Lond. B Biol. Sci..

[210] Carrasco-Gil S., Rodríguez-Menéndez S., Fernández B., Pereiro R., de La Fuente V., Hernandez-Apaolaza L. (2018). Silicon induced Fe deficiency affects Fe, Mn, Cu and Zn distribution in rice (*Oryza sativa* L.) growth in calcareous conditions. Plant Physiol. Biochem..

[211] Barisone G.A., O’Donnell R.T., Ma Y., Abuhay M.W., Lundeberg K., Gowda S., Tuscano J.M. (2018). A purified, fermented, extract of *Triticum aestivum* has lymphomacidal activity mediated via natural killer cell activation. PLoS ONE.

[212] Coskun D., Deshmukh R., Sonah H., Menzies J.G., Reynolds O., Ma J.F., Kronzucker H.J., Bélanger R.R. (2019). The controversies of silicon’s role in plant biology. New Phytol..

[213] Kaur H., Greger M. (2019). A Review on Si Uptake and Transport System. Plants.

[214] Azad M.O.K., Park B.S., Adnan M., Germ M., Kreft I., Woo S.H., Park C.H. (2021). Silicon biostimulant enhances the growth characteristics and fortifies the bioactive compounds in common and Tartary buckwheat plant. J. Crop Sci. Biotechnol..

[215] Kaynar O., Berktas Y. (2010). How to Choose the Right Plate for Thin-Layer Chromatography?. Rev. Anal. Chem..

[216] Urbain A., Simões-Pires C.A., Meyers R.A. (2006). Thin-layer Chromatography of Plants, with Chemical and Biological Detection Methods. Encyclopedia of Analytical Chemistry.

[217] Mathias E., Halkar U. (2004). Separation and characterization of lignin compounds from the walnut (Juglans regia) shell oil using preparative TLC, GC–MS and 1H NMR. J. Anal. Appl. Pyrolysis.

[218] Grzelak E.M., Hwang C., Cai G., Nam J.-W., Choules M.P., Gao W., Lankin D.C., McAlpine J.B., Mulugeta S.G., Napolitano J.G. (2016). Bioautography with TLC-MS/NMR for Rapid Discovery of Anti-tuberculosis Lead Compounds from Natural Sources. ACS Infect. Dis..

[219] Kisomi A.S., Alizadeh T., Shakeri A., Nouri A., Farsadrooh M., Najafi AsliPashaki S. (2020). Application of μ-TLC for speciation of inorganic arsenic by laser ablation inductively coupled plasma mass spectrometry. Microchem. J..

[220] Vorapalawut N., Martinez Labrador M., Pohl P., Caetano M., Chirinos J., Arnaudguilhem C., Bouyssiere B., Shiowatana J., Lobinski R. (2012). Application of TLC and LA ICP SF MS for speciation of S, Ni and V in petroleum samples. Talanta.

[221] Rezić I., Špehar M., Jakovljević S. (2017). Characterization of Ag and Au nanolayers on Cu alloys by TLC, SEM-EDS, and ICP-OES. Mater. Corros..

[222] Rodrigues F.Á., McNally D.J., Datnoff L.E., Jones J.B., Labbé C., Benhamou N., Menzies J.G., Bélanger R.R. (2004). Silicon Enhances the Accumulation of Diterpenoid Phytoalexins in Rice: A Potential Mechanism for Blast Resistance. Phytopathology.

[223] Sytar O., Bośko P., Živčák M., Brestic M., Smetanska I. (2018). Bioactive Phytochemicals and Antioxidant Properties of the Grains and Sprouts of Colored Wheat Genotypes. Molecules.

[224] Bélanger J.M., Jocelyn Paré J.R., Sigouin M. (1997). Chapter 2 High performance liquid chromatography (HPLC): Principles and applications. Instrumental Methods in Food Analysis.

[225] Rahman M., Sarker S.D., Nahar L. (2018). Application of computational methods in isolation of plant secondary metabolites. Computational Phytochemistry.

[226] Ponce de León C.A., Montes-Bayón M., Caruso J.A. (2002). Elemental speciation by chromatographic separation with inductively coupled plasma mass spectrometry detection. J. Chromatogr. A.

[227] Lech K., Jarosz M. (2011). Novel methodology for the extraction and identification of natural dyestuffs in historical textiles by HPLC–UV–Vis–ESI MS. Case study: Chasubles from the Wawel Cathedral collection. Anal. Bioanal. Chem..

[228] Chawla G., Ranjan C. (2016). Principle, Instrumentation, and Applications of UPLC: A Novel Technique of Liquid Chromatography. Open Chem. J..

[229] Batool A., Menaa F. (2020). Concentration and purification of seaweed components by chromatography methods. Sustainable Seaweed Technologies.

[230] Rainville P.D., Theodoridis G., Plumb R.S., Wilson I.D. (2014). Advances in liquid chromatography coupled to mass spectrometry for metabolic phenotyping. Trends Anal. Chem..

[231] Taleuzzaman M., Ali S., Gilani S., Imam S., Hafeez A. (2015). Ultra Performance Liquid Chromatography (UPLC)—A Review. Austin J. Anal. Pharm. Chem..

[232] Vega I., Rumpel C., Ruíz A., La Mora M.d.L., Calderini D.F., Cartes P. (2020). Silicon Modulates the Production and Composition of Phenols in Barley under Aluminum Stress. Agronomy.

[233] Williams R.J.P., Evered D., O’Connor M. (2007). Introduction to Silicon Chemistry and Biochemistry. Ciba Foundation Symposium 121—Silicon Biochemistry.

[234] Fleck A.T., Nye T., Repenning C., Stahl F., Zahn M., Schenk M.K. (2011). Silicon enhances suberization and lignification in roots of rice (*Oryza sativa*). J. Exp. Bot..

[235] Vivancos J., Labbé C., Menzies J.G., Bélanger R.R. (2015). Silicon-mediated resistance of Arabidopsis against powdery mildew involves mechanisms other than the salicylic acid (SA)-dependent defence pathway. Mol. Plant Pathol..

[236] Karpagasundari C., Kulothungan S. (2014). Analysis of bioactive compounds in *Physalis minima* leaves using GC MS, HPLC, UV-VIS and FTIR techniques. J. Pharmacogn. Phytochem..

[237] Chavan S.S., Jadhav R.S., Khemanar K.S., Tambe V.B. (2015). Evaluation of Antibacterial Activity and Phytochemical Screening of *Medicago sativa* Leaves. Int. J. Curr. Res. Acad. Rev..

[238] Saniewska A., Jurzysta M., Biały Z. (2001). Differential antifungal activity of alfalfa (*Medicago santva* L.) saponins originated from roots and aerial parts for some ornamental plant pathogens. Acta Agrobot..

[239] D’Addabbo T., Argentieri M.P., Żuchowski J., Biazzi E., Tava A., Oleszek W., Avato P. (2020). Activity of Saponins from *Medicago* Species against Phytoparasitic Nematodes. Plants.

[240] Saeed S., Tariq P. (2005). Antibacterial activities of *Mentha piperita*, *Pisum sativum* and *Momordica charantia*. Pak. J. Bot..

[241] Wang H.X., Ng T.B. (2006). An antifungal protein from the pea *Pisum sativum* var. arvense Poir. Peptides.

[242] Ye X., Ng T. (2003). Isolation of pisumin, a novel antifungal protein from legumes of the sugar snap pea *Pisum sativum* var. macrocarpon. Comp. Biochem. Physiol. C Pharmacol. Toxicol. Pharmacol..

[243] Rehman S., Khanum A. (2011). Isolation and Characterization of Peptide (S) from *Pisum sativum* Having Antimicrobial Activity against Various Bacteria. Pak. J. Bot..

[244] Erecevit P., Kırbağ S. (2017). Determining the Phytochemical Parameters of *Pisum sativum* (pease) and Effects on the Development of *Debaryomyces hansenii*. Indian J. Pharm. Educ. Res..

[245] De Caleya R.F., Gonzalez-Pascual B., García-Olmedo F., Carbonero P. (1972). Susceptibility of Phytopathogenic Bacteria to Wheat Purothionins In Vitro. Appl. Microbiol..

[246] Caruso C., Caporale C., Chilosi G., Vacca F., Bertini L., Magro P., Poerio E., Buonocore V. (1996). Structural and antifungal properties of a pathogenesis-related protein from wheat kernel. J. Protein Chem..

[247] Broekaert W.F., van Parijs J., Allen A.K., Peumans W.J. (1988). Comparison of some molecular, enzymatic and antifungal properties of chitinases from thorn-apple, tobacco and wheat. Physiol. Mol. Plant Pathol..

[248] Saha S., Islam Z., Islam S., Hossain M., Islam S.M. (2018). Evaluation of antimicrobial activity of wheat (*Triticum aestivum* L.) against four bacterial strains. SKUAST J. Res..

[249] Rajpurohit L., Mehta N., Ankola A., Gadiyar A. (2015). Evaluation of the anti-microbial activity of various concentration of wheat grass (*Triticum aestivum*) extract against Gram-positive bacteria: An in vitro study. J. Dent. Res. Rev..

[250] Schalchli H., Pardo F., Hormazábal E., Palma R., Guerrero J., Bensch E. (2012). Antifungal activity of wheat root exudate extracts on *Gaeumannomyces graminis* var. Tritici growth. J. Soil Sci. Plant Nutr..

[251] Sharma N., Tiwari V., Vats S., Kumari A., Chunduri V., Kaur S., Kapoor P., Garg M. (2020). Evaluation of Anthocyanin Content, Antioxidant Potential and Antimicrobial Activity of Black, Purple and Blue Colored Wheat Flour and Wheat-Grass Juice against Common Human Pathogens. Molecules.

[252] Rajoria A., Mehta A., Mehta P., Ahirwal L., Shukla S. (2015). Phytochemical analysis and estimation of major bioactive compounds from *Triticum aestivum* L. grass with antimicrobial potential. Pak. J. Pharm. Sci..

[253] Choma I., Jesionek W. (2015). TLC-Direct Bioautography as a High Throughput Method for Detection of Antimicrobials in Plants. Chromatography.

[254] Borisov R., Kanateva A., Zhilyaev D. (2021). Recent Advances in Combinations of TLC With MALDI and Other Desorption/Ionization Mass-Spectrometry Techniques. Front. Chem..

[255] Kroslakova I., Pedrussio S., Wolfram E. (2016). Direct Coupling of HPTLC with MALDI-TOF MS for Qualitative Detection of Flavonoids on Phytochemical Fingerprints. Phytochem. Anal..

[256] Cebolla V.L., Jarne C., Vela J., Garriga R., Membrado L., Galbán J. (2021). Scanning densitometry and mass spectrometry for HPTLC analysis of lipids: The last 10 years. J. Liq. Chromatogr. Relat. Technol..

[257] Bañuelos-Hernández A.E., Azadniya E., Ramírez Moreno E., Morlock G.E. (2020). Bioprofiling of *Mexican Plectranthus amboinicus* (Lour.) essential oil via planar chromatography–effect-directed analysis combined with direct analysis in real time high-resolution mass spectrometry. J. Liq. Chromatogr. Relat. Technol..

[258] Abouzeid S., Beutling U., Elekhnawy E., Selmar D. (2023). Antibacterial and Antibiofilm Effects of Allelopathic Compounds Identified in Medicago sativa L. Seedling Exudate against Escherichia coli. Molecules.

[259] Chamachar M.M., Fazeli M.R., Salimi M., Samadi N. (2022). Growth promoting activity, anti-biofilm effect, and down regulation of papC and rcsA genes expression by *Medicago sativa* (alfalfa) extract. Food Biosci..

[260] Silva N.B.S., Marques L.A., Röder D.D.B. (2020). Antibiofilm Activity of Natural Products: Promising Strategies for Combating Microbial Biofilms. Ann. Trop. Med. Public Health.

[261] Zainab M.H., Zainab S.H., Sraa N.M., Elaf S.M., Hala M.S. (2022). Antibiofilm formation activity of purified lectin from wheat seeds (*Triticum aestivum*) against *Candida albicans* causing oral candidiasis. Tex. J. Multidiscip. Stud..

[262] González-Ortiz G., van Quarles Ufford H.C., Halkes S.B.A., Cerdà-Cuéllar M., Beukelman C.J., Pieters R.J., Liskamp R.M.J., Pérez J.F., Martín-Orue S.M. (2014). New properties of wheat bran: Anti-biofilm activity and interference with bacteria quorum-sensing systems. Environ. Microbiol..

[263] Gatouillat G., Alabdul Magid A., Bertin E., Okiemy-Akeli M.-G., Morjani H., Lavaud C., Madoulet C. (2014). Cytotoxicity and Apoptosis Induced by Alfalfa (*Medicago sativa*) Leaf Extracts in Sensitive and Multidrug-Resistant Tumor Cells. Nutr. Cancer.

[264] Gatouillat G., Magid A.A., Bertin E., El btaouri H., Morjani H., Lavaud C., Madoulet C. (2015). Medicarpin and millepurpan, two flavonoids isolated from *Medicago sativa*, induce apoptosis and overcome multidrug resistance in leukemia P388 cells. Phytomedicine.

[265] Avato P., Migoni D., Argentieri M., Fanizzi F.P., Tava A. (2017). Activity of Saponins from Medicago species Against HeLa and MCF-7 Cell Lines and their Capacity to Potentiate Cisplatin Effect. Anticancer Agents Med. Chem..

[266] Lanza A., Tava A., Catalano M., Ragona L., Singuaroli I., Della Robustelli Cuna F.S., Della Robustelli Cuna G. (2004). Effects of the Medicago scutellata trypsin inhibitor (MsTI) on cisplatin-induced cytotoxicity in human breast and cervical cancer cells. Anticancer Res..

[267] El-Feky A.M., Elbatanony M.M., Mounier M.M. (2018). Anti-cancer potential of the lipoidal and flavonoidal compounds from *Pisum sativum* and *Vicia faba* peels. Egypt. J. Basic Appl. Sci..

[268] Stanisavljević N.S., Ilić M.D., Matić I.Z., Jovanović Ž.S., Čupić T., Dabić D.Č., Natić M.M., Tešić Ž.L. (2016). Identification of Phenolic Compounds from Seed Coats of Differently Colored European Varieties of Pea (*Pisum sativum* L.) and Characterization of Their Antioxidant and In Vitro Anticancer Activities. Nutr. Cancer.

[269] Abd El-Galil A.A., Negm El-Dein A., Awad H.M., Helmy W.A. (2021). Chemical composition and biological activities of aqueous extracts and their sulfated derivatives of pea peel (*Pisum sativum* L.). Biocatal. Agric. Biotechnol..

[270] Khalaf Z.A. (2012). Extraction and Purification of Asparaginase Enzyme from *Pisum sativum* Plant and Studying Their Cytotoxicity against L20B Tumor Cell Line. Master’s Thesis.

[271] El-Aassar M.R., Hafez E.E., El-Deeb N.M., Fouda M.M. (2014). Microencapsulation of lectin anti-cancer agent and controlled release by alginate beads, biosafety approach. Int. J. Biol. Macromol..

[272] Patel A. (2014). Isolation, characterization and production of a new recombinant lectin protein from leguminous plants. Bio. Chem. Comp..

[273] Clemente A., Gee J.M., Johnson I.T., MacKenzie D.A., Domoney C. (2005). Pea (*Pisum sativum* L.) Protease Inhibitors from the Bowman−Birk Class Influence the Growth of Human Colorectal Adenocarcinoma HT29 Cells *in Vitro*. J. Agric. Food Chem..

[274] Vincentini O., Maialetti F., Gazza L., Silano M., Dessi M., de Vincenzi M., Pogna N.E. (2007). Environmental factors of celiac disease: Cytotoxicity of hulled wheat species *Triticum monococcum*, *T. turgidum* ssp. dicoccum and *T. aestivum* ssp. *spelta*. J. Gastroenterol. Hepatol..

[275] Rajoria A., Mehta A., Mehta P., Ahirwal L., Shukla S., Bajpai V.K. (2017). Evaluation of antiproliferative and hepatoprotective effects of wheat grass (*Triticum aestivum*). Acta Biol. Hung..

[276] Patel J.B. (2016). Anticancer & Cytotoxic potential of aqueous extract of *Triticum aestivum* on HeLa cell line. J. Drug Deliv. Ther..

[277] Patel J.B., Patel P., Parmar R.S., Patel D. (2019). Anticancer and Cytotoxic Potential of Aqueous Extract of Triticum aestivum on Colorectal Carcinoma. J. Drug Deliv. Ther..

[278] Zagórska-Dziok M., Ziemlewska A., Nizioł-Łukaszewska Z., Bujak T. (2020). Antioxidant Activity and Cytotoxicity of *Medicago sativa* L. Seeds and Herb Extract on Skin Cells. BioResearch Open Access.

[279] Clemente A., Carmen Marín-Manzano M., Jiménez E., Carmen Arques M., Domoney C. (2012). The anti-proliferative effect of TI1B, a major Bowman–Birk isoinhibitor from pea (*Pisum sativum* L.), on HT29 colon cancer cells is mediated through protease inhibition. Br. J. Nutr..

[280] Tillmann M.T., Mendes C.B.D.M., Fischer G., Varela Júnior A.S., Fernandes C.G., Nobre M.D.O. (2018). *Triticum aestivum* in open skin wounds: Cytotoxicity and collagen histopathology. SCA.

